# Supplementary material for: Identification of Freezing-Responsive microRNAs and Their Targets in Chinese Jujube by Small RNA and Degradome Sequencing
Source: Int J Mol Sci. 2026 Sep 4;27(17):7912. doi: 10.3390/ijms27177912 (PMC13566890; doi:10.3390/ijms27177912)
Supplement: Supplementary file 1 [file ijms-27-07912-s001.zip › Supplementary Material S2/novel_miR_135.pdf]

Provisional ID	: novel_miR_135
Score total	: 205993.6
Score for star read(s)	: 3.9
Score for read counts	: 205987.3
Score for mfe	: 0.8
Score for randfold	: 1.6
Score for cons. seed	:
Total read count	: 404047
Mature read count	: 396583
Loop read count	: 14
Star read count	: 7450

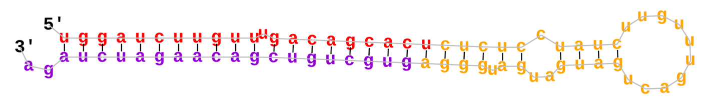

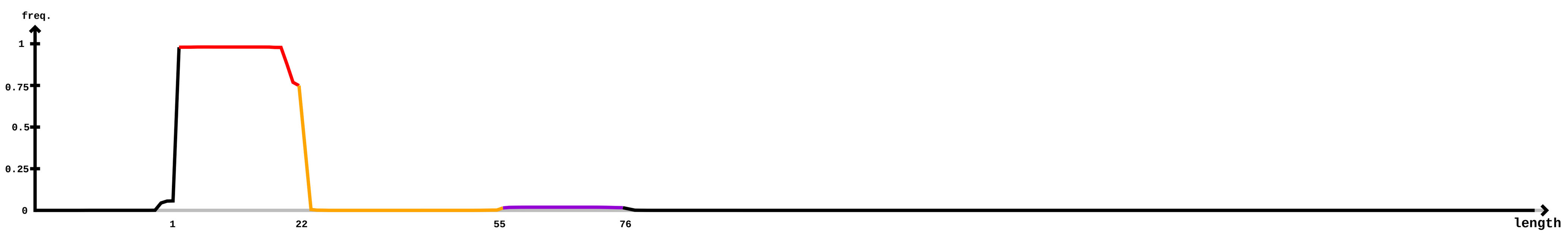

Mature Star

```
5'- uccaucuuguucguuuacccucuggaucuuguuugacagcacucucuccuaucuuguuugacugaugaugaugggga gugcugucgacaagaucuagaagguacugucgaauaagauacagauacggaggaaggaucaacuuuaccuacucucacacaaucuucuucauauucucuucaauuuuuauuuuuuuauuguucguauuuuuuuucccagauuagauuucccuguuaaucaccaugguauuaaca -3' obs
    uccaucuuguucguuuacccucuggaucuuguuugacagcacucucuccuaucuuguuugacugaugaugaugggagugcugucgacaagaucuagaagguacugucgaauaagauacagauacggaggaaggaucaacuuuaccuacucucacacaaucuucuucauauucucuucaauuuuuauuuuuuuauuguucguauuuuuuuucccagauuagauuucccuguuaaucaccaugguauuaaca exp
    ...(((((((((..((((.((((((((((((.(((((((((((((.((((.........))))..)).))))))))))))))))))))))))).))))....))))))))))..(((((((((((((.........................)))))))..........((((............))))..)))))))......................(((((((.((....))))))))) reads mm sample
```

	reads	mm	sample
.ccaucuuguucguuuacc...	1	0	LT-1
.ccaucuuguucguuuacccucugg...	5	0	LCK-3
.ccaucuuguucguuuacccucuggauc...	1	0	LT-3
...aucuuguucguuuacccucugga...	2	0	JCK-1
.....cuuguucguuuacccucuggauc...	4	0	LT-2
......uuguucguuuaccUucuggauc...	3	1	JCK-3
......uuguucguuuaccUucuggauc...	2	1	JCK-1
......uuguucguuuaccUucuggauc...	6	1	JCK-2
......uuguucguuuaccUucuggauc...	2	1	LT-2
.......uguucguuuacccucuggauc...	2	0	JCK-3
.......uguucguuuacccucuggauc...	1	0	JCK-1
.......uguucguuuaccUucuggauc...	10	1	JCK-1
.......uguucguuuaccUucuggauc...	3	1	LT-2
.......uguucguuuaccUucuggauc...	31	1	JCK-2
.......uguucguuuaccUucuggauc...	12	1	LT-1
.......uguucguuuaccUucuggauc...	30	1	JCK-3
.......uguucguuuacccucuggauc...	1	0	JCK-2
.......uguucguuuaccUucuggauc...	12	1	LT-3
..........ucguuuacccucuggaucuug...	1	0	LT-2
............guuuacccucuggaucuuguuugaca...	2	0	LCK-3
.............uuuacccucuggaucuuguA...	1	1	JCK-1
.............uuuacccucuggaucuuguuu...	2	0	JCK-3
.............uuuacccucuggaucuuguuuga...	1	0	JCK-1
...............uacccucuggaucuuguuugacagc...	2	0	JCK-2
................Ccccucuggaucuuguuuga...	1	1	LT-1
................acccucuggaucuuguuugacagc...	2	0	JCK-2
................acccucuggaucuuguuugacagca...	2	0	JCK-2
................acccucuggaucuuguuugacagca...	2	0	JCK-3
.................cccucuggaucuuguuugacagc...	1	0	LT-3
..................ccucuggaucuuguuugac...	3	0	JCK-2
..................ccucuggaucuuguuugaca...	2	0	JCK-3
..................ccucuggaucuuguuugaca...	2	0	JCK-1
..................ccucuggaucuuguuugacag...	4	0	LCK-3

Mature Star

uccaucuuguucguuuacccucuggaucuuguuugacagcacucucuccuaucuuguuugacugaugaugaugggagugcugucgacaagaucuagaagguacugucgaauaagauacagauacggaggaaggaucaacuuuaccuacucucacacaaucuucuucauauucucuucaauuuuauuuuuuuauuguucguauuuuuuuucccagauuagauuucccuguuaaucaccaugguauuaaca

..................ccucuggaucuuguuugacag	1	0	JCK-3
..................ccucuggaucuuguuugacag	1	0	JCK-2
..................ccucuggaucuuguuugacag	1	0	JCK-1
..................ccucuggaucuuguuugacagc	32	0	JCK-2
..................ccucuggaucuuguuugacagc	6	0	LT-1
..................ccucuggaucuuguuugacagc	6	0	LCK-3
..................ccucuggaucuuguuugacagc	21	0	JCK-1
..................ccucuggaucuuguuugacagc	3	0	LT-2
..................ccucuggaucuuguuugacagc	35	0	JCK-3
..................ccucuggaucuuguuugacagc	9	0	LT-3
..................ccucuggaucuuguuugacagcU	4	1	JCK-2
..................ccucuggaucuuguuugacagcU	2	1	JCK-1
..................ccucuggaucuuguuugacagcU	1	1	JCK-3
...................cucuggaucuuguuugac	13	0	LT-2
...................cucuggaucuuguuugac	10	0	JCK-3
...................cucuggaucuuguuugac	13	0	JCK-2
...................cucuggaucuuguuugac	4	0	JCK-1
...................cucuggaucuuguuugac	8	0	LT-1
...................cucuggaucuuguuugaU	2	1	JCK-3
...................cucuggaucuuguuugac	6	0	LCK-3
...................cucuggCucuuguuugac	1	1	JCK-2
...................cucuggaucuuguuugac	13	0	LT-3
...................cucuggaucuuguuugaca	41	0	JCK-2
...................cuNuggaucuuguuugaca	1	1	LT-2
...................cucuggCucuuguuugaca	1	1	LCK-1
...................cucuggaucuuguuugacU	6	1	JCK-3
...................cucuggaucuuguuugaca	20	0	JCK-1
...................cucuggaucuuguuugacU	14	1	LCK-3
...................cucuggaucuuguuugacC	1	1	LT-1
...................cucuggaucuuguuugaca	62	0	LT-2
...................cucuggaucuuguuugaUa	1	1	JCK-3
...................cucuggaucuuguuugaca	28	0	JT-1
...................cucuggaucuuguuugaUa	1	1	JCK-2
...................cucuggaucuuguuugaca	21	0	JT-3
...................cucuggaucuuguuugaca	65	0	LT-3
...................cucuggaucuuguuugacU	1	1	LT-3
...................cucuggaucuuguuugaca	78	0	LCK-3
...................cucuggCucuuguuugaca	1	1	JT-1
...................cucuggaucuuguuugaca	18	0	JT-2
...................cucuggaucuuguuugaca	42	0	LCK-1
...................cucuggaucuuguuugaca	27	0	LT-1
...................cucuggaucuuguuugaca	29	0	LCK-2
...................cucuggaucuuguuugacU	7	1	JCK-2
...................cucuggaucuuguuugacU	6	1	LT-2
...................cucuggaucuuguuugacU	2	1	LT-1
...................cucuggaucuuguuugaca	18	0	JCK-3
...................cucuggaucuuguuugacag	7	0	LT-3
...................cucuggaucuuguuugacag	7	0	LT-1
...................cucuggaucuuguuugacag	14	0	JT-2
...................cucuggaucuuguuugacag	15	0	JT-3
...................cucuggaucuuguuugacag	12	0	LT-2
...................cucuggaucuuguuugacag	6	0	JCK-3
...................cucuggaucuuguuugacaU	2	1	LT-1
...................cucuggaucuuguuugacag	23	0	LCK-3
...................cucuggaucuuguuugacag	4	0	JCK-2
...................cucuggaucuuAuuugacag	1	1	LT-2
...................cucuggaucuuguuugacag	5	0	JCK-1
...................cucuggaucuuguuugacaU	3	1	LT-3
...................cucuggCucuuguuugacagc	4	1	JCK-3
...................cucuggCucuuguuugacagc	11	1	LT-3
...................cucuggCucuuguuugacagc	1	1	JCK-1
...................cCcuggaucuuguuugacagc	1	1	LCK-2
...................cNcuggaucuuguuugacagc	1	1	JCK-3
...................cucuggaucuuguuugacagc	1675	0	LT-2
...................cucuggaucuuguCugacagc	1	1	JCK-2
...................cucuggaucuuguuugaGagc	1	1	JCK-1
...................cucuggaucuuguCugacagc	1	1	JCK-3
...................cucuggCucuuguuugacagc	20	1	LT-2
...................cucuggaucuuNuuugacagc	3	1	JCK-1
...................cucuggaCcuuguuugacagc	2	1	LT-1

Mature Star

```
uccaucuuguucguuuacccucuggaucuuguuugacagcacucucuccuaucuuguuugacugaugaugauggagugcugucgacaagaucuagaagguacugucgaauaagauacagauacggaggaaggaucaacuuuaccuacucucacacaaucuucuucauauucucuucaauuuuuauuuuuuuauuguucguauuuuuuuucccagauuagauuucccuguuaaucaccaugguauuaaca
```

Read	Count	Mismatch	Sample
...................cucuAgaucuuguuugacagc	1	1	JCK-1
...................cCcuggaucuuguuugacagc	2	1	LT-2
...................cucuggaucuuAuuugacagc	1	1	LT-1
...................cucuggaucuuguuugCcagc	4	1	JCK-1
...................cGcuggaucuuguuugacagc	1	1	LT-2
...................cucuggaucuuguuugacCgc	2	1	JCK-2
...................cucuggCucuuguuugacagc	11	1	LT-1
...................cucuggaucuuguuugacCgc	2	1	JCK-3
...................cucuUgaucuuguuugacagc	1	1	LT-3
...................cucuAgaucuuguuugacagc	1	1	LCK-3
...................cucuggaucuuguuugacGgc	1	1	LT-3
...................cucCggaucuuguuugacagc	3	1	LT-3
...................cucuggauUuuguuugacagc	1	1	LT-1
...................cNcuggaucuuguuugacagc	2	1	JCK-1
...................cucuggaucuCguuugacagc	2	1	LT-2
...................cuUuggaucuuguuugacagc	3	1	JCK-3
...................cucuggaucuuguuugacagc	1680	0	JCK-1
...................cucuggaucuCguuugacagc	1	1	LT-1
...................cucuggaucuuUuuugacagc	1	1	LT-1
...................cucuggCucuuguuugacagc	2	1	LCK-1
...................cucCggaucuuguuugacagc	1	1	LT-2
...................cucNggaucuuguuugacagc	2	1	JCK-1
...................cucuggaucuuAuuugacagc	1	1	LT-3
...................cucuggaucuuguuugCcagc	5	1	LT-3
...................cucuggaucuuguuugacCgc	2	1	LCK-3
...................cucuggaucuuguuuAacagc	1	1	LT-1
...................cucuggaucuuguuugaUagc	1	1	LT-3
...................cCcuggaucuuguuugacagc	1	1	JCK-2
...................cucuggauAuuguuugacagc	1	1	LT-1
...................cucuggaucuugCuugacagc	1	1	JCK-2
...................cucuggaucuuguuugaUagc	2	1	JCK-3
...................cucuggaucuuguuuAacagc	1	1	LCK-3
...................cucuggCucuuguuugacagc	4	1	LCK-3
...................cucuggaucuuguuugGcagc	1	1	JCK-3
...................cNcuggaucuuguuugacagc	4	1	JCK-2
...................cucuggaucuuguuugCcagc	2	1	LCK-3
...................cucCggaucuuguuugacagc	1	1	LT-1
...................cucuggauNuuguuugacagc	2	1	JCK-3
...................cucuggaucuuguuugacagc	62	0	JT-2
...................cucuggaucuuguuugacagc	66	0	JT-1
...................cuUuggaucuuguuugacagc	2	1	JCK-1
...................cucuggaucuuguuugaUagc	2	1	JCK-2
...................cucuggaucCuguuugacagc	1	1	LT-1
...................cNcuggaucuuguuugacagc	1	1	LCK-1
...................cucuggaucuuguuugacagc	1379	0	LCK-3
...................cucugAaucuuguuugacagc	1	1	LT-3
...................cuNuggaucuuguuugacagc	2	1	LT-2
...................cucuggaucuuguuugacagc	2907	0	JCK-2
...................cucuggaucuuguuugCcagc	1	1	JCK-3
...................cucuggCucuuguuugacagc	1	1	LCK-2
...................cucCggaucuuguuugacagc	2	1	JCK-3
...................cucuggaucuuguuugacagU	2	1	LT-3
...................cNcuggaucuuguuugacagc	1	1	LT-3
...................cuAuggaucuuguuugacagc	2	1	JCK-3
...................cucuggaucuuguuugCcagc	3	1	JCK-2
...................cucuggaucuuguuugacagc	3319	0	LT-1
...................cucuggaucuuguuugacagc	3001	0	LT-3
...................cucuggaucuuguuugacCgc	2	1	LT-2
...................cucugUaucuuguuugacagc	1	1	LT-1
...................cucuggaucuuguuugacagc	292	0	LCK-2
...................cucCggaucuuguuugacagc	2	1	JCK-2
...................cucuggaucuCguuugacagc	1	1	LCK-3
...................cucuggaucuuAuuugacagc	2	1	JCK-2
...................cCcuggaucuuguuugacagc	1	1	LCK-3
...................cucuggaucuuguuugacagU	1	1	JCK-3
...................cucuggaucuuUuuugacagc	1	1	JCK-3
...................cucugNaucuuguuugacagc	1	1	LCK-1
...................cucuggaucuuguuugacCgc	2	1	JCK-1
...................cucGggaucuuguuugacagc	1	1	JCK-1
...................cucuggaucCuguuugacagc	1	1	JCK-2

```
                   Mature                                    Star
uccaucuuguucguuuacccucuggaucuuguuugacagcacucucuccuaucuuguuugacugaugaugaugggagugcugucgacaagaucuagaagguacugucgaauaagauacagauacggaggaaggaucaacuuuaccuacucucacacaaucuucuucauauucucuucaauuuuuauuuuuuuauuguucguauuuuuuuucccagauuagauuucccuguuaaucaccaugguauuaaca
...................cucugAaucuuguuugacagc..............................   3     1   LT-2
...................cuGuggaucuuguuugacagc..............................   1     1   LT-2
...................cucuggaucuuguuugCcagc..............................   7     1   LT-2
...................cucuggaucuuguuugacCgc..............................   2     1   LT-1
...................cucuAgaucuuguuugacagc..............................   4     1   LT-2
...................cucuggaucuuguuugCcagc..............................   9     1   LT-1
...................cucuggaucuuguuugacagc..............................   275   0   LCK-1
...................cucuAgaucuuguuugacagc..............................   3     1   LT-3
...................cucuggaucuuguuugacaNc..............................   4     1   JCK-1
...................cCcuggaucuuguuugacagc..............................   1     1   LT-1
...................cucuggCucuuguuugacagc..............................   11    1   JCK-2
...................cucuggaucuuguuuUacagc..............................   1     1   JCK-2
...................cucuggaucuuguuugacagA..............................   3     1   LT-2
...................cucuUgaucuuguuugacagc..............................   1     1   LT-2
...................cucuggaucuuguuugacagc..............................   90    0   JT-3
...................cucuAgaucuuguuugacagc..............................   1     1   JCK-2
...................cucuggaucuuguuugacCgc..............................   4     1   LT-3
...................cucuggaucuuguuugacagc..............................   2088  0   JCK-3
...................cucugAaucuuguuugacagc..............................   1     1   JCK-3
...................cucuggaucuuguuugacagcU.............................   13    1   JT-2
...................cucuggaucuuguuugacagca.............................   22    0   LT-3
...................cucuggaucuugCuugacagca.............................   1     1   JCK-2
...................cucuggaucuuguuugacagca.............................   5     0   LCK-3
...................cucuggaucuuguuugacagcU.............................   55    1   JCK-2
...................cucuggaucuuguuugacagcC.............................   1     1   JCK-2
...................cucuggaucuuguuugacagcG.............................   1     1   JT-2
...................cucuggaucuuguuugacagca.............................   19    0   LT-2
...................cucuggaucuuguuugacagcU.............................   47    1   LT-2
...................cucuggaucuuguuugacagcU.............................   14    1   JT-3
...................cucuggaucuuguuugacagcU.............................   25    1   JCK-1
...................cucuggaucuuguuugacagcC.............................   2     1   LT-1
...................cucuggaucuuguuugacagcU.............................   84    1   LCK-3
...................cucuggaucuuguuugacagca.............................   72    0   JCK-1
...................cucuggaucuuguuugacagca.............................   117   0   JCK-2
...................cucuggaucuuguuugacagcU.............................   29    1   JCK-3
...................cucuggaucuuguuugacagcU.............................   66    1   LT-1
...................cucuggaucuuguuugacagcU.............................   69    1   LT-3
...................cucuggaucuuguuugacagcC.............................   2     1   LT-2
...................cucuggaucuuguuugacagca.............................   10    0   JT-3
...................cucuggaucuuguuugacagca.............................   87    0   JCK-3
...................cucuggaucuuguuugacagca.............................   1     0   JT-2
...................cucuggaucuuguuugacagca.............................   27    0   LT-1
...................cucuggaucuuguuuNacagca.............................   1     1   JCK-3
...................cucuggaucuuguuugacagcC.............................   2     1   LT-3
...................cucuggaucuuguuugacagcac............................   1     0   LT-3
...................cucuggaucuuguuugacagcac............................   1     0   LT-1
...................cucuggaucuuguuugacagcacN...........................   1     1   LT-2
...................cucuggaucuuguuugacagcacu...........................   1     0   LT-2
....................ucuggaucuuguuugacU................................   6     1   LT-1
....................ucuggaucuuguuugaca................................   3     0   LCK-2
....................ucuggaucuuguuugaca................................   28    0   LT-1
....................ucuggaucuuguuugaca................................   33    0   JCK-2
....................ucuggaucuuguuugaca................................   9     0   JT-1
....................ucuggaucuuguuugaca................................   41    0   LT-2
....................ucuggaucuuguuugaca................................   25    0   JCK-1
....................ucuggaucuuguuugaca................................   26    0   LT-3
....................ucuggaucuuguuugaca................................   30    0   JT-2
....................ucuggaucuuguuugaca................................   35    0   JT-3
....................ucuggaucuuguuugaca................................   79    0   LCK-3
....................ucuggaucuuguuugaca................................   3     0   LCK-1
....................ucuggaucuuguuugacU................................   1     1   JCK-2
....................ucuggaucuuguuugacU................................   2     1   LT-3
....................ucuggaucuuguuugacU................................   1     1   JCK-1
....................ucuggaucuuguuugacU................................   9     1   LCK-3
....................ucuggaucuuguuugaca................................   15    0   JCK-3
....................ucuggaucuuguuugacag...............................   2     0   LT-1
....................ucuggCucuuguuugacagc..............................   2     1   LT-2
....................ucuggaucuuguuugacagc..............................   107   0   LT-1
....................ucuggaucuuguuugacagc..............................   29    0   JCK-1
....................ucuggaucuuguuuUacagc..............................   1     1   LCK-2
```

Mature Star

uccaucuuguucguuuacccucuggaucuuguuugacagcacucucuccuaucuuguuugacugaugaugauggagugcugucgacaagaucuagaagguacugucgaauaagauacagauacggaggaaggaucaacuuuaccuacucucacacaaucuucuucauauucucuucaauuuuuauuuuuuuauuguucguauuuuuuuucccagauuagauuucccuguuaaucaccaugguauuaaca

....................ucuggaucuuguuugacagc	63	0	LCK-1
....................ucuggaucuuguuugacagc	127	0	LT-3
....................ucuggaucuuguuugacagc	75	0	LCK-2
....................ucuggaucuuguuugacagA	1	1	LT-3
....................ucuggaucuuguuuNacagc	1	1	LCK-1
....................ucuggaucuuguuugacagc	35	0	JCK-3
....................ucuggaucuuguuugacagc	116	0	LT-2
....................ucuggaucuuguuugacagA	1	1	JCK-3
....................ucuggaucuuguuugacaUc	1	1	LT-1
....................ucuggaucuuguuugacagc	55	0	JCK-2
....................ucuggaucuuguuugacagc	41	0	LCK-3
....................ucuggaucuuguuugacagA	1	1	JCK-1
....................uGuggaucuuguuugacagc	1	1	LCK-1
....................ucuggCucuuguuugacagc	1	1	LCK-1
....................ucCggaucuuguuugacagca	1	1	JCK-2
....................ucuggaucuuguuugacagca	12	0	JT-3
....................ucuggaucuuguuugUcagca	1	1	JCK-3
....................ucuggaucuuguuugacaUca	3	1	JCK-3
....................ucuggaucuuguuugacagca	267	0	LT-3
....................ucuggaucuuguuugacagcU	6	1	LT-1
....................ucuggaucuuguuugacaUca	5	1	JCK-2
....................ucuggauUuuguuugacagca	1	1	JCK-2
....................ucuggaucuuguuugCcagca	1	1	LT-1
....................ucuggaucuuguuugCcagca	2	1	LT-3
....................ucuggaucuuguuugacagcU	6	1	JCK-1
....................ucuggaucuuguuugacagca	145	0	LCK-3
....................ucuggaucuuguuugacagNa	2	1	JCK-1
....................ucuggaucuuguuugCcagca	1	1	JCK-1
....................uUuggaucuuguuugacagca	1	1	JCK-1
....................ucuggaucuCguuugacagca	1	1	LT-3
....................ucuggaucuuguuugacagca	29	0	JT-1
....................ucuggauAuuguuugacagca	1	1	JCK-2
....................uUuggaucuuguuugacagca	12	1	JCK-2
....................ucuggaucuuguuugacagcU	3	1	JCK-3
....................ucuggaucuuguuugUcagca	1	1	JCK-2
....................ucuggaucuuguuugacagcU	3	1	JCK-2
....................uUuggaucuuguuugacagca	8	1	JCK-3
....................ucuggaucuuguuugacagca	17	0	LCK-2
....................ucuggaucuuguuugacagcN	1	1	JCK-1
....................ucuggaucuugGuugacagca	2	1	LT-1
....................ucuggaucuuguuugacagcU	14	1	LT-2
....................ucuggaucuuguuugacagca	299	0	LT-1
....................ucuggaucuuguuugacagca	11	0	JT-2
....................uNuggaucuuguuugacagca	1	1	JCK-1
....................ucuggCucuuguuugacagca	1	1	JCK-2
....................ucuggaucuuguuugacagca	1097	0	JCK-2
....................ucuggaucuugGuugacagca	1	1	JCK-1
....................ucuggaucuuguuugacagca	195	0	LT-2
....................ucuggaucuuguuugacagca	9	0	LCK-1
....................ucuggaucCuguuugacagca	1	1	JCK-2
....................ucuggaucuuguuugacGgca	2	1	JCK-1
....................ucCggaucuuguuugacagca	1	1	JCK-1
....................ucuggaucuuguuugGcagca	1	1	LT-2
....................ucuggaucuuguuugacagcU	9	1	LCK-3
....................ucuggaucuuguuugacagca	628	0	JCK-1
....................ucuggaucuuguuugacagcC	1	1	LCK-3
....................ucuggaucuuguuugacagca	832	0	JCK-3
....................ucuNgaucuuguuugacagca	1	1	JCK-1
....................ucuggaucuuguuugGcagca	1	1	LCK-3
....................ucuggaucuuguuugacagUa	1	1	JCK-2
....................ucuggaucuuguuugacagcU	7	1	LT-3
....................ucuggaucuuguuugacaUca	2	1	JCK-1
....................ucuggaucuuguuugacagcac	27	0	LT-1
....................ucuggaucuuguuugCcagcac	1	1	LT-2
....................ucuggaucuuguuugacagcac	17	0	LT-2
....................ucuggaucuuguuugacagcac	22	0	JCK-3
....................ucuggaucuuguuugacagcac	50	0	JCK-2
....................ucuggaucuuguuugacagcac	19	0	LCK-3
....................ucuggaucuuguuugacagcaU	1	1	LT-1
....................ucuggaucuuguuugacagcaU	2	1	LT-2

Mature **Star**

```
uccaucuuguucguuuacccucuggaucuuguuugacagcacucucuccuaucuuguuugacugaugaugaugggagugcugucgacaagaucuagaagguacugucgaauaagauacagauacggaggaaggaucaacuuuaccuacucucacacaaucuucuucauauucucuucaauuuuuauuuuuuuauuguucguauuuuuuuucccagauuagauuucccuguuaaucaccaugguauuaaca
.....................ucuggaucuuguuugacagcac      24    0   LT-3
.....................ucuggaucuuguuugacagcac      34    0   JCK-1
.....................ucuggaucuuguuugacagcacu     2     0   JCK-3
.....................ucuggaucuuguuugacagcacu     2     0   JCK-1
.....................ucuggaucuuguuugacagcacu     3     0   LT-1
.....................ucuggaucuuguuugacagcacu     5     0   JCK-2
.....................ucuggaucuuguuugacagcaUu     1     1   LT-3
.....................ucuggaucuuguuugacagcacuc    1     0   JCK-1
.....................ucuggaucuuguuugacagcacuc    2     0   LT-3
.....................ucuggaucuuguuugacagcacuc    1     0   JCK-2
.....................ucuggaucuuguuugacagcacuc    1     0   JCK-3
.....................ucuggaucuuguuugacagcacucu   2     0   LT-1
......................cuggaucuuguuugacagc        13    0   LT-2
......................cuggaucuuguuugacagc        5     0   LT-1
......................cuggaucuuguuugacagc        1     0   LT-3
......................cuggaucuuguuugacagc        5     0   LCK-3
......................cuggaucuuguuugacagc        2     0   JCK-3
......................cuggaucuuguuugacagc        7     0   JCK-2
......................cuggaucuuguuugacagc        1     0   JCK-1
......................cuggaucuuguuugacagca       15    0   LT-3
......................cuggaucuuguuugacagcU       1     1   JCK-2
......................cuggaucuuguuugacagcU       1     1   LT-3
......................cuggaucuuguuugacagcU       4     1   JT-2
......................cuggaucuuguuugacagca       58    0   JCK-1
......................cuggaucuuguuugacagca       61    0   JCK-2
......................cuggaucuuguuugacagcU       5     1   LT-2
......................cuggaucuuguuugacagca       11    0   LT-2
......................cuggaucuuguuugacagca       51    0   JCK-3
......................cuggaucuuguuugacagcU       4     1   JT-3
......................cuggaucuuguuugCcagca       1     1   JCK-1
......................cuggaucuuguuugacagca       12    0   LCK-3
......................cuggaucuuguuugacagcU       1     1   JCK-3
......................cuggaucuuguuugacagca       9     0   LT-1
......................cuggaucuuguuugacagcac      3     0   LT-3
......................cuggaucuuguuugacagcac      4     0   LT-2
......................cuggaucuuguuugacagcac      1     0   LCK-3
......................cuggaucuuguuugacagcacu     13    0   LT-3
......................cuggaucuuguuugacagcaUu     2     1   JCK-3
......................cuggaucuuguuugacagcacu     15    0   LCK-3
......................cuggaucuuguuugacagcacu     46    0   JCK-2
......................cuggaucuuguuugacagcacu     22    0   JCK-3
......................cuggaucuuguuugacagcacu     17    0   JCK-1
......................cuggaucuuguuugacagcacu     4     0   LT-1
......................cuggaucuuguuugacagcacu     7     0   LT-2
......................cuggaucuuguuugacagcaUu     1     1   JCK-2
......................cuggaucuuguuugacagcacuc    1     0   JCK-2
......................cuggaucuuguuugacagcacuc    1     0   JCK-1
......................cuggaucuuguuugacagcacuc    3     0   JCK-3
......................cuggaucuuguuugacagcacucu   3     0   JCK-2
......................cuggaucuuguuugacagcacucuc  1     0   JCK-1
......................cuggaucuuguuugacagcacucuc  3     0   JCK-2
.......................uggaucuuguuuNacagc        3     1   LCK-1
.......................uggaucuuguuugacCgc        1     1   JT-1
.......................uggaucuuguuugacagc        1810  0   JCK-2
.......................uggCucuuguuugacagc        2     1   LCK-1
.......................uggCucuuguuugacagc        10    1   LCK-3
.......................uggCucuuguuugacagc        2     1   JT-2
.......................uUgaucuuguuugacagc        1     1   LT-3
.......................uggaucuuguuugacagc        1080  0   JCK-1
.......................uggCucuuguuugacagc        3     1   JT-1
.......................ugAaucuuguuugacagc        1     1   LT-1
.......................uggaucuuguuugacagU        9     1   LT-1
.......................uggaucuugCuugacagc        1     1   JCK-1
.......................uAgaucuuguuugacagc        1     1   LT-1
.......................uggCucuuguuugacagc        3     1   LCK-2
.......................uggaucuuguuuAacagc        1     1   LCK-2
.......................uggaucuuguuugacagc        1447  0   LCK-1
.......................uggaucuuguuugacagc        419   0   JT-3
.......................uggCucuuguuugacagc        2     1   JT-3
.......................uggNucuuguuugacagc        1     1   JCK-1
```

```
                     Mature                                                Star
uccaucuuguucguuuacccucuggaucuuguuugacagcacucucuccuaucuuguuugacugaugaugaugggagugcugucgacaagaucuagaagguacugucgaauaagauacagauacggaggaaggaucaacuuuaccuacucucacacaaucuucuucauauucucuucaauuuuuauuuuuuuauuguucguauuuuuuuucccagauuagauuucccuguuaaucaccaugguauuaaca
......................uggaucuuguuugacagU..................................................................................................................................................................................  21    1  LT-2
......................ugAaucuuguuugacagc..................................................................................................................................................................................  3     1  LCK-3
......................uggaucuuguuugacagc..................................................................................................................................................................................  2328  0  LT-3
......................uggaucuuAuuugacagc..................................................................................................................................................................................  1     1  LT-3
......................uggCucuuguuugacagc..................................................................................................................................................................................  5     1  LT-1
......................uggaucuuguuugacCgc..................................................................................................................................................................................  2     1  LCK-3
......................uggaucuuguuuUacagc..................................................................................................................................................................................  1     1  JT-2
......................uggaucuuguuugCcagc..................................................................................................................................................................................  4     1  LT-3
......................uggaucuuAuuugacagc..................................................................................................................................................................................  3     1  LT-2
......................uggaucuuguuugacagA..................................................................................................................................................................................  4     1  LCK-3
......................uggaucuuguuugacagA..................................................................................................................................................................................  1     1  JCK-3
......................uNgaucuuguuugacagc..................................................................................................................................................................................  1     1  JCK-1
......................uggCucuuguuugacagc..................................................................................................................................................................................  1     1  JCK-3
......................uggaucuuAuuugacagc..................................................................................................................................................................................  1     1  JCK-1
......................uggaucuuguuugacagc..................................................................................................................................................................................  520   0  JT-1
......................ugAaucuuguuugacagc..................................................................................................................................................................................  1     1  JCK-2
......................uggaucuuguuugacagG..................................................................................................................................................................................  1     1  JCK-1
......................uggaucuuguuNgacagc..................................................................................................................................................................................  1     1  JCK-1
......................uggaucuuguuNgacagc..................................................................................................................................................................................  1     1  JCK-2
......................ugUaucuuguuugacagc..................................................................................................................................................................................  1     1  LT-2
......................uggaucuuguuugCcagc..................................................................................................................................................................................  6     1  LT-1
......................uggaucuuguuugCcagc..................................................................................................................................................................................  4     1  JCK-2
......................uggaucuuguuugacagU..................................................................................................................................................................................  3     1  JCK-2
......................uggaucuuguuugacagc..................................................................................................................................................................................  1573  0  LCK-2
......................uggCucuuguuugacagc..................................................................................................................................................................................  2     1  JCK-2
......................uggaucuuguuugGcagc..................................................................................................................................................................................  2     1  LCK-1
......................uggaucuuguuNgacagc..................................................................................................................................................................................  1     1  JT-1
......................uggaucuuguuugaNagc..................................................................................................................................................................................  11    1  LCK-1
......................uggaucuuguuugacaCc..................................................................................................................................................................................  2     1  JCK-3
......................uAgaucuuguuugacagc..................................................................................................................................................................................  1     1  LCK-2
......................uggaucuuguuugCcagc..................................................................................................................................................................................  2     1  LCK-2
......................uggaucuuAuuugacagc..................................................................................................................................................................................  1     1  LCK-2
......................Cggaucuuguuugacagc..................................................................................................................................................................................  1     1  LT-2
......................uggaucuuguuugacCgc..................................................................................................................................................................................  2     1  LCK-2
......................uggaucuuguuugCcagc..................................................................................................................................................................................  1     1  JCK-3
......................uCgaucuuguuugacagc..................................................................................................................................................................................  1     1  LT-1
......................uggaucuuguGugacagc..................................................................................................................................................................................  1     1  LT-2
......................uggauNuuguuugacagc..................................................................................................................................................................................  1     1  LCK-1
......................uggaucuuguuugacagA..................................................................................................................................................................................  7     1  LT-2
......................uggaucuuguuugacagc..................................................................................................................................................................................  6351  0  LCK-3
......................uggaucuuguuugCcagc..................................................................................................................................................................................  2     1  LCK-1
......................uggaucuuguuugacagU..................................................................................................................................................................................  1     1  JCK-3
......................uggaucuugCuugacagc..................................................................................................................................................................................  1     1  LT-1
......................uggGucuuguuugacagc..................................................................................................................................................................................  1     1  LT-2
......................ugAaucuuguuugacagc..................................................................................................................................................................................  1     1  LT-3
......................ugAaucuuguuugacagc..................................................................................................................................................................................  2     1  LCK-1
......................ugNaucuuguuugacagc..................................................................................................................................................................................  1     1  LCK-1
......................Cggaucuuguuugacagc..................................................................................................................................................................................  1     1  LT-3
......................uAgaucuuguuugacagc..................................................................................................................................................................................  4     1  LCK-3
......................uggaucuuguuugacagA..................................................................................................................................................................................  3     1  JCK-1
......................uNgaucuuguuugacagc..................................................................................................................................................................................  3     1  JCK-2
......................uAgaucuuguuugacagc..................................................................................................................................................................................  4     1  LT-3
......................uAgaucuuguuugacagc..................................................................................................................................................................................  5     1  LT-2
......................uggCucuuguuugacagc..................................................................................................................................................................................  53    1  LT-2
......................uggaucuAguuugacagc..................................................................................................................................................................................  1     1  LT-1
......................uggaucuuguuugNcagc..................................................................................................................................................................................  6     1  LCK-1
......................uggaucuuguuugacCgc..................................................................................................................................................................................  2     1  LT-1
......................uggaucuuguuugacCgc..................................................................................................................................................................................  2     1  JCK-2
......................ugUaucuuguuugacagc..................................................................................................................................................................................  1     1  JCK-1
......................uggaucuuguuugacagU..................................................................................................................................................................................  12    1  LT-3
......................uggCucuuguuugacagc..................................................................................................................................................................................  1     1  JCK-1
......................uggaucuuguuugaGagc..................................................................................................................................................................................  4     1  LCK-1
......................uggaucuuguuuAacagc..................................................................................................................................................................................  1     1  LCK-3
......................uggaucuuguuugacCgc..................................................................................................................................................................................  6     1  LT-2
......................uUgaucuuguuugacagc..................................................................................................................................................................................  1     1  LT-2
......................uUgaucuuguuugacagc..................................................................................................................................................................................  1     1  JCK-2
......................uggNucuuguuugacagc..................................................................................................................................................................................  1     1  JT-2
......................uNgaucuuguuugacagc..................................................................................................................................................................................  1     1  LT-3
......................uNgaucuuguuugacagc..................................................................................................................................................................................  1     1  JT-1
......................uNgaucuuguuugacagc..................................................................................................................................................................................  2     1  LCK-1
```

Mature Star

uccaucuuguucguuuacccucuggaucuuguuugacagcacucucuccuaucuuguuugacugaugaugaugggagugcugucgacaagaucuagaagguacugucgaauaagauacagauacggaggaaggaucaacuuuaccuacucucacacaaucuucuucauauucucuucaauuuuuauuuuuuuauuguucguauuuuuuuucccagauuagauuucccuguuaaucaccaugguauuaaca			
......................uggaucuuguuugacagA......	6	1	LT-3
......................uggaucuuguuugacagc......	2286	0	LT-1
......................uggaucuCguuugacagc......	1	1	LT-1
......................ugNaucuuguuugacagc......	7	1	LT-2
......................uggaucuuguuugacagc......	5296	0	LT-2
......................uggaucuuguuugCcagc......	15	1	LCK-3
......................uUgaucuuguuugacagc......	1	1	LCK-3
......................ugAaucuuguuugacagc......	3	1	LT-2
......................ugUaucuuguuugacagc......	1	1	LCK-3
......................uggaucuuguuugCcagc......	3	1	JCK-1
......................uggaucuuguuugacagU......	1	1	JCK-1
......................uggaucuuguuugacGgc......	1	1	LT-2
......................uggaucuuguuugacagU......	13	1	LCK-3
......................uggaucCuguuugacagc......	1	1	LT-3
......................uggaucuuAuuugacagc......	3	1	LT-1
......................uggaucuugCuugacagc......	1	1	JCK-2
......................uggaucuuguCugacagc......	2	1	JCK-2
......................uggCucuuguuugacagc......	7	1	LT-3
......................uggaucuuguuugacagc......	1255	0	JCK-3
......................uNgaucuuguuugacagc......	1	1	JCK-3
......................uggGucuuguuugacagc......	1	1	LCK-3
......................uggaucuuguuugacagA......	1	1	LCK-1
......................uggaucuuguuugCcagc......	24	1	LT-2
......................uNgaucuuguuugacagc......	1	1	LCK-3
......................uggaucuuguuugacagc......	353	0	JT-2
......................uggauUuuguuugacagca......	1	1	LT-2
......................uggaucuuguuugacagcU......	735	1	JT-1
......................uggaucuuguuugacCgca......	2	1	JT-3
......................uggaucuuguuugacCgca......	2	1	LT-3
......................uggGucuuguuugacagca......	1	1	JCK-1
......................uggaucuuUuuugacagca......	1	1	LCK-2
......................uggaucuuguuugacagcU......	1214	1	JCK-2
......................uggaucuuguuugaAagca......	1	1	LCK-3
......................uggCucuuguuugacagca......	2	1	LT-1
......................uggaucuuguuGgacagca......	9	1	LCK-3
......................uNgaucuuguuugacagca......	3	1	JT-1
......................uggaucuuguuugacagcU......	2081	1	LT-3
......................uggaucuuguuugacagca......	3633	0	LCK-2
......................uggaucuuguuugNcagca......	9	1	LCK-1
......................uggaucuuguuugacagcC......	14	1	LT-1
......................uggaucuuguuugacagcG......	15	1	LT-2
......................uggaucuuguuugacagcU......	4465	1	LCK-3
......................uggaucuuguuugacCgca......	1	1	LT-2
......................uggaucuuguuugacagcU......	835	1	JCK-3
......................uggCucuuguuugacagca......	3	1	JT-2
......................uggaucuuguuugacCgca......	2	1	LCK-1
......................uggaucuCguuugacagca......	1	1	LCK-2
......................uggaucCuguuugacagca......	1	1	LT-3
......................uggaucuuguuugacagca......	3565	0	LCK-1
......................uggauNuuguuugacagca......	10	1	LCK-1
......................uggaucuuguuugCcagca......	6	1	LCK-2
......................uggaucuuguuugacagAa......	1	1	LT-3
......................uggCucuuguuugacagca......	2	1	LT-3
......................uggCucuuguuugacagca......	4	1	LCK-3
......................uUgaucuuguuugacagca......	5	1	JT-3
......................uggaucuuguuuGcagca......	1	1	JT-2
......................uggaucuuguuugacagca......	1294	0	JT-3
......................uNgaucuuguuugacagca......	1	1	LCK-3
......................uggaucuuguuugCcagca......	3	1	LCK-1
......................uggaucuuguuugacagca......	980	0	JT-2
......................uggaucuuguuugacagcU......	1354	1	LT-1
......................uggaucuuguuugacagUa......	24	1	JCK-2
......................uggaucuuguuugacagcC......	7	1	LCK-1
......................uggCucuuguuugacagca......	2	1	JCK-1
......................uggaucuuguuugacagcG......	11	1	LCK-3
......................uggCucuuguuugacagca......	7	1	LCK-2
......................uggaucuuguuugacagcG......	6	1	JCK-1
......................uAgaucuuguuugacagca......	1	1	JT-1
......................uAgaucuuguuugacagca......	3	1	LCK-3
......................uggaucuugCuugacagca......	1	1	JT-1

Mature Star

uccaucuuguucguuuacccucuggaucuuguuugacagcacucucuccuaucuuguuugacugaugaugaugggagugcugucgacaagaucuagaagguacugucgaauaagauacagauacggaggaaggaucaacuuuaccuacucucacacaaucuucuucauauucucuucaauuuuuauuuuuuuauuguucguauuuuuuuucccagauuagauuucccuguuaaucaccaugguauuaaca			
......................uggaucuuguuugacagcC..	21	1	LT-3
......................uggaucuuguuugacCgca..	3	1	LCK-3
......................uggaucuuguuugCcagca..	2	1	LT-3
......................uggaucuuguuugacagUa..	1	1	LCK-2
......................uggaucuuguuNgacagca..	1	1	JT-2
......................uggaucuuguuugacCgca..	1	1	JT-1
......................uggaucuuguuugacagcG..	2	1	JCK-3
......................uggaucuuguuugacagcC..	20	1	LT-2
......................uggaucuuguuugacagUa..	18	1	LCK-3
......................uggaucuuguuugacagUa..	11	1	JCK-3
......................uggaucuuguuugacagcG..	2	1	JT-2
......................uggCucuuguuugacagca..	2	1	JT-3
......................ugAaucuuguuugacagca..	1	1	LT-3
......................uggCucuuguuugacagca..	5	1	LCK-1
......................uggaucuuguuugacagca..	1105	0	JCK-3
......................uNgaucuuguuugacagca..	1	1	JCK-3
......................uggaucuuguuugacagca..	3109	0	LCK-3
......................uggaucuuguuugaAagca..	1	1	LCK-2
......................uggaucuuguuugacagUa..	10	1	JT-3
......................uggaucuuguuugacagUa..	11	1	LT-3
......................uggaucCuguuugacagca..	1	1	LCK-1
......................uggaucuuguuugacagUa..	3	1	LT-1
......................uggaucuuguuugacagcC..	5	1	JCK-2
......................uggaucuuguuugacagcU..	279	1	JT-2
......................uggaucuuguuugacagcC..	6	1	JCK-3
......................uggaucuugNuugacagca..	1	1	LCK-1
......................uggaucuuguuugacagcU..	1372	1	LCK-1
......................uUgaucuuguuugacagca..	5	1	JT-2
......................uggaucuuguuugacagca..	1154	0	JCK-1
......................uggaucuuguuugacagcU..	2784	1	LT-2
......................uggaucuuguuugacagcU..	1503	1	LCK-2
......................uggaucuuguuugCcagca..	5	1	JCK-2
......................uggaucuuguuugacagcG..	11	1	LT-3
......................uNgaucuuguuugacagca..	2	1	JCK-2
......................uggaucCuguuugacagca..	1	1	LT-2
......................uAgaucuuguuugacagca..	1	1	LT-3
......................uggCucuuguuugacagca..	4	1	JCK-2
......................uggaucuuguuugacagcC..	2	1	JCK-1
......................uggaucuuguuNgacagca..	5	1	JCK-1
......................uggaucuuguuugacagca..	1862	0	JCK-2
......................uggaucuuguuugCcagca..	2	1	LCK-3
......................uggaucuuguuNgacagca..	2	1	JT-1
......................uggaucuuguuugacagcU..	381	1	JT-3
......................uggaucuuguuugacagca..	2333	0	LT-2
......................uggaucuCguuugacagca..	1	1	LT-1
......................uggaucuuguuugacagUa..	29	1	LT-2
......................uggGucuuguuugacagca..	1	1	LCK-2
......................uggaucGuguuugacagca..	1	1	JT-3
......................uggaucuuguuugacagcC..	21	1	LCK-3
......................uAgaucuuguuugacagca..	1	1	LT-2
......................ugAaucuuguuugacagca..	1	1	JT-1
......................uggaucuuguuugacCgca..	1	1	JCK-1
......................uggaucuuguuugacagcC..	3	1	JT-1
......................ugNaucuuguuugacagca..	9	1	LT-2
......................ugUaucuuguuugacagca..	1	1	LCK-2
......................uggaucuuguuuUacagca..	1	1	LCK-3
......................uggaucuuguuugGcagca..	1	1	LT-2
......................uggaucuuguuugGcagca..	2	1	LCK-1
......................uggCucuuguuugacagca..	5	1	JT-1
......................uggaucuCguuugacagca..	1	1	LT-2
......................uggaucuuguuugaNagca..	11	1	LCK-1
......................uggaucuuguuugacCgca..	1	1	JCK-3
......................uggaucuuguuugCcagca..	2	1	JCK-1
......................uggaucuuguuugCcagca..	1	1	LT-1
......................Cggaucuuguuugacagca..	1	1	LCK-3
......................uggaucuuguuugacagcU..	718	1	JCK-1
......................uggaucuuguuugacagca..	1490	0	LT-3
......................uggaucuuguuugacagcC..	8	1	LCK-2
......................uggaucuCguuugacagca..	1	1	JT-1
......................uggCucuuguuugacagca..	3	1	JCK-3

Mature Star

```
uccaucuuguucguuuacccucuggaucuuguuugacagcacucucuccuaucuuguuugacugaugaugaugggagugcugucgacaagaucuagaagguacugucgaauaagauacagauacggaggaaggaucaacuuuaccuacucucacacaaucuucuucauauucucuucaauuuuuauuuuuuuauuguucguauuuuuuuucccagauuagauuucccuguuaaucaccaugguauuaaca
......................uggauAuuguuugacagca......  1     1  JCK-3
......................uggaucuuguuugacagcG......  2     1  JCK-2
......................uggaucuuguuugacagUa......  12    1  JCK-1
......................uggaCcuuguuugacagca......  1     1  LT-2
......................uggaucuuguuuNacagca......  2     1  LCK-1
......................ugAaucuuguuugacagca......  1     1  JCK-3
......................uggaucuuguuugacagcG......  12    1  LT-1
......................uggaucuuAuuugacagca......  1     1  LCK-2
......................uggCucuuguuugacagca......  12    1  LT-2
......................uggaucuuAuuugacagca......  1     1  LCK-3
......................uggaucGuguuugacagca......  1     1  LCK-2
......................uggaucuuguuugacagUa......  11    1  JT-2
......................uggaucuuguuugacagca......  858   0  LT-1
......................uAgaucuuguuugacagca......  1     1  LCK-2
......................uUgaucuuguuugacagca......  1     1  LT-3
......................uggaucuuguuugaGagca......  4     1  LCK-1
......................uggaucuuguuugCcagca......  11    1  LT-2
......................uggaucuuguuugacagca......  1426  0  JT-1
......................uNgaucuuguuugacagca......  2     1  JCK-1
......................uggNucuuguuugacagca......  1     1  LCK-1
......................uggaucuuguuugacCgca......  2     1  JCK-2
......................ugNaucuuguuugacagca......  5     1  LCK-1
......................uAgaucuuguuugacagcac......  1     1  LT-1
......................uggaucuuguuugacagcac......  666   0  LT-1
......................uggaucuuguCugacagcac......  1     1  JCK-2
......................uggGucuuguuugacagcac......  2     1  LT-2
......................uggaucuuguuugCcagcac......  3     1  JCK-2
......................uggCucuuguuugacagcac......  1     1  LT-1
......................uggaucuuguuugCcagcac......  1     1  JCK-1
......................uggaucuuguuugacagcac......  545   0  LCK-1
......................uggaucuuguuugCcagcac......  1     1  JCK-3
......................uggaucuuguuugacagcaU......  16    1  JT-1
......................uggaucuuCuuugacagcac......  1     1  LCK-3
......................uggaucuuguuugacagcaU......  22    1  LT-1
......................uggaucuuguuugacagcUc......  77    1  LT-2
......................uggaucuuguuugCcagcac......  1     1  LCK-3
......................uggaucuuguuugacagcUc......  26    1  LCK-1
......................uAgaucuuguuugacagcac......  4     1  LT-2
......................uggaucuuguuugacagcac......  715   0  LT-3
......................uggaucuuguuugacagcac......  249   0  JT-3
......................uggaucuuUuuugacagcac......  1     1  LT-3
......................uggaucuugCuugacagcac......  1     1  LT-2
......................uggaucuuguuugacagcUc......  31    1  LCK-2
......................uggaucuuguuugCcagcac......  2     1  JT-1
......................uggaucuuguuNgacagcac......  1     1  JT-2
......................uggaucuuguuugacCgcac......  3     1  LT-3
......................uggaucuuguuugacagUac......  1     1  JCK-3
......................uggaCcuuguuugacagcac......  1     1  JT-1
......................Aggaucuuguuugacagcac......  1     1  LCK-3
......................uggCucuuguuugacagcac......  5     1  LCK-3
......................uggaucuuguuugacagcac......  982   0  LT-2
......................uggaucuuguuugacagcUc......  51    1  LT-3
......................uggaucuuguuugCcagcac......  4     1  LT-2
......................uggaucuuguuugacagcaU......  26    1  LT-3
......................uggaucuuguuugacagcaA......  1     1  LCK-2
......................uggaucuuguuugGcagcac......  1     1  LT-3
......................uNgaucuuguuugacagcac......  1     1  LCK-3
......................uAgaucuuguuugacagcac......  1     1  LCK-3
......................uggaucuuguuugacagcac......  525   0  LCK-2
......................uggaucuuguuugacagcaA......  1     1  JCK-2
......................uggaucuuguuugacagcaA......  4     1  JCK-3
......................uggaucuuguuuAacagcac......  1     1  LCK-2
......................uggaucuuguuugacagcaU......  8     1  JCK-1
......................ugNaucuuguuugacagcac......  3     1  LT-2
......................uggaucuuguuugacagcaU......  46    1  JCK-2
......................uggaucuuguuugacagcaU......  26    1  JCK-3
......................uggaucuuguuugacagcaA......  1     1  JT-3
......................uggCucuuguuugacagcac......  2     1  LT-3
......................uggauNuuguuugacagcac......  1     1  LCK-1
......................uggaucuuguuugacagcaU......  66    1  LCK-3
```

Mature **Star**

uccaucuuguucguuuacccucuggaucuuguuugacagcacucucuccuaucuuguuugacugaugaugaugggagugcugucgacaagaucuagaagguacugucgaauaagauacagauacggaggaaggaucaacuuuaccuacucucacacaaucuucuucauauucucuucaauuuuuauuuuuuuauuguucguauuuuuuuucccagauuagauuucccuguuaaucaccaugguauuaaca			
......................uNgaucuuguuugacagcac	1	1	JCK-1
......................uggaucuuguuugCcagcac	1	1	JT-2
......................uggaucuuguuugNcagcac	2	1	LCK-1
......................uggaucuuguuugacagcaU	26	1	LT-2
......................uggaucuuguuugacagcCc	2	1	LT-1
......................uggaucuuguuugacagcUc	6	1	JCK-1
......................ugAaucuuguuugacagcac	1	1	JCK-1
......................uggaucuuguuugacagcNc	1	1	JCK-1
......................uggaucuuguuugacagcUc	15	1	JCK-2
......................uggCucuuguuugacagcac	9	1	LT-2
......................uggCucuuguuugacagcac	1	1	LCK-1
......................uggaucuuguuugacagcaG	1	1	LT-1
......................uggaucuuguuugacagcac	216	0	JT-1
......................uggaCcuuguuugacagcac	1	1	JCK-2
......................uggCucuuguuugacagcac	2	1	JCK-2
......................uggaucuuguuugacagcCc	1	1	JT-1
......................uggaucuuguuugacagcac	586	0	JCK-1
......................uggaucuuguuugacagcac	1318	0	LCK-3
......................uggaucuuguuugCcagcac	1	1	LT-1
......................uggCucuuguuugacagcac	1	1	JCK-1
......................uggaucuuguuugacagcUc	7	1	JCK-3
......................uggaucuuguuugacCgcac	1	1	LT-2
......................uggaucuuguuugGcagcac	1	1	LCK-1
......................uAgaucuuguuugacagcac	1	1	LCK-1
......................uggaucuuguuugacagcCc	1	1	LT-2
......................uggaucuuguuugacGgcac	1	1	LT-2
......................uggaCcuuguuugacagcac	1	1	JCK-3
......................uggaucuuguuugacagcac	899	0	JCK-2
......................uggCucuuguuugacagcac	1	1	LCK-2
......................uggaucuuguuugacagcac	687	0	JCK-3
......................uggaucuuguuugacagcUc	63	1	LCK-3
......................uggaucuuguuugacagcaA	3	1	LT-3
......................uggaucuuguuugacCgcac	2	1	JCK-2
......................uggaucuuguuugacagcGc	1	1	LCK-3
......................uggaucuuguuugacagcUc	36	1	LT-1
......................uggCucuuguuugacagcac	2	1	JT-3
......................uggaucuuguuugacagcaA	3	1	JCK-1
......................uggaucuuguuugacagcaN	2	1	JCK-1
......................uggaucuuguuugacagcac	170	0	JT-2
......................uggaucuuguuugCcagcac	1	1	LT-3
......................uggaucuuguuugaNagcac	2	1	LCK-1
......................uggaucuuguuugCcagcacu	61	1	LT-1
......................uggaucuuguuugCcagcacu	5	1	LCK-1
......................uAgaucuuguuugacagcacu	8	1	JCK-2
......................uggaucuuguuuUacagcacu	4	1	LT-1
......................uggaucuuguuugacagcaAu	4	1	LT-2
......................uggaucuuguuugacagGacu	2	1	JCK-3
......................uggaucuuguCugacagcacu	3	1	LT-1
......................uggauUuuguuugacagcacu	4	1	JCK-3
......................uNgaucuuguuugacagcacu	2	1	LT-3
......................uggaucuuguuugacagAacu	1	1	JCK-2
......................uggaucuuguuGgacagcacu	1	1	JCK-3
......................uggaucuuguuugacCgcacu	26	1	LT-2
......................uggGucuuguuugacagcacu	8	1	JCK-1
......................uggaucuuguuugacagUacu	5	1	LT-3
......................uggauUuuguuugacagcacu	3	1	JCK-1
......................uggaucuCguuugacagcacu	5	1	JCK-1
......................uggaucuuguuugacCgcacu	12	1	LT-1
......................uggauNuuguuugacagcacu	1	1	JCK-2
......................uggaucuuguuugacagcCcu	4	1	LCK-1
......................ugAaucuuguuugacagcacu	19	1	JCK-2
......................uggaucuuguuugGcagcacu	1	1	LT-2
......................uggaCcuuguuugacagcacu	4	1	LT-3
......................uggaucuuguuugacagUacu	13	1	JCK-3
......................ugAaucuuguuugacagcacu	3	1	JCK-1
......................uggauUuuguuugacagcacu	1	1	LT-2
......................uggaucuuguuugacagcacG	12	1	LT-3
......................uggGucuuguuugacagcacu	8	1	JCK-2
......................uggaucuuguuugacagAacu	1	1	JCK-1
......................uggaucuugAuugacagcacu	1	1	JCK-2

Mature **Star**

uccaucuuguucguuuacccucuggaucuuguuugacagcacucucuccuaucuuguuugacugaugaugaugggagugcugucgacaagaucuagaagguacugucgaauaagauacagauacggaggaaggaucaacuuuaccuacucucacacaaucuucuucauauucucuucaauuuuuauuuuuuuauuguucguauuuuuuuucccagauuagauuucccuguuaaucaccaugguauuaaca

uggaucuuguuuAacagcacu	6	1	JCK-1
uUgaucuuguuugacagcacu	7	1	LT-2
uggaucuuUuuugacagcacu	1	1	LT-3
uggaucuuguuugacagcaUu	22	1	JT-2
uggaucuuguuugacagcacG	1	1	JT-2
uggaucuuguuugacagcacu	23834	0	LT-1
uggaucuuguCugacagcacu	1	1	LT-2
ugUaucuuguuugacagcacu	3	1	LT-3
ugUaucuuguuugacagcacu	2	1	JCK-1
uggaucuuguuugaNagcacu	17	1	LCK-1
uggaCcuuguuugacagcacu	13	1	JCK-3
uggaucuuguuNgacagcacu	1	1	JT-1
uggaucCuguuugacagcacu	2	1	LT-2
uggaucuuguuugacagcaUu	20	1	LT-1
uggaucuuguuugacagcacN	22	1	JCK-1
uggaucuuguuugNcagcacu	15	1	LCK-1
uggaucuugGuugacagcacu	1	1	JCK-1
uggaucuuguuugacagcacG	2	1	LCK-1
uggaucuuguuugacagcacu	658	0	JT-2
uggaucuuguuugacagcaUu	1732	1	JCK-3
uggaucuuguuugacagcCcu	34	1	LT-2
uggaucuuguuuUacagcacu	3	1	LT-3
Cggaucuuguuugacagcacu	11	1	JCK-2
uggaucuuguuugacagUacu	34	1	JCK-2
ugCaucuuguuugacagcacu	2	1	LT-2
uggNucuuguuugacagcacu	1	1	LCK-1
uggaucuuguuugacGgcacu	7	1	JCK-1
uggaucuugCuugacagcacu	4	1	LCK-3
uggaucuugGuugacagcacu	1	1	JCK-2
uNgaucuuguuugacagcacu	104	1	JCK-2
uggCucuuguuugacagcacu	118	1	JCK-3
ugUaucuuguuugacagcacu	1	1	JCK-2
uggaucuuguuugacagcacG	7	1	LCK-3
ugAaucuuguuugacagcacu	4	1	LCK-3
uggaucuugCuugacagcacu	6	1	JCK-2
uggaucCuguuugacagcacu	2	1	LT-3
uggaucuuguuAgacagcacu	1	1	JCK-1
uggCucuuguuugacagcacu	7	1	JT-1
uggaucuuguuugacagcaAu	10	1	JCK-2
uggaucuuCuuugacagcacu	1	1	JCK-2
uggaucuuguuugaAagcacu	2	1	JCK-1
uggaucuuguuugacagcUcu	1	1	JCK-2
uggaucuuguuugacCgcacu	3	1	LCK-1
uggauNuuguuugacagcacu	3	1	LCK-1
uggaucuuUuuugacagcacu	2	1	LT-1
uggCucuuguuugacagcacu	39	1	LCK-3
uggaucuuguuugacagcaUu	68	1	LT-2
uggauAuuguuugacagcacu	2	1	JCK-2
uggaucuuguuugacagcaUu	2361	1	JCK-2
uggaucuuAuuugacagcacu	20	1	JCK-2
uggaucuCguuugacagcacu	1	1	LT-1
uggaucuuguuNgacagcacu	121	1	JCK-1
uggaucuuguGugacagcacu	2	1	LT-2
uggaucCuguuugacagcacu	10	1	JCK-1
uggaucuuguuugacagcacG	10	1	LT-1
uggaucuuguuugacCgcacu	5	1	LCK-2
uggaucuuguuugacagcaUu	64	1	LT-3
uggaCcuuguuugacagcacu	2	1	LT-2
uggaucuuguuugacagcaAu	5	1	JT-2
uggaucuuguuugacagcaAu	3	1	LT-3
uggaucuuguuugacGgcacu	2	1	LT-1
Cggaucuuguuugacagcacu	6	1	JCK-1
uggaucuuguuugacagcGcu	3	1	LT-2
uggaucuuguuugacagcCcu	4	1	LCK-2
ugAaucuuguuugacagcacu	2	1	LCK-1
uggaucuuguuuUacagcacu	2	1	LT-2
uggaucuugCuugacagcacu	4	1	LT-2
uggaucuuAuuugacagcacu	9	1	LT-2
uggaucuuguuugCcagcacu	86	1	JCK-1
uggaucuuguuugGcagcacu	1	1	LT-3

```
                      Mature                                                     Star
uccaucuuguucguuuacccucuggaucuuguuugacagcacucucuccuaucuuguuugacugaugaugaugggagugcugucgacaagaucuagaagguacugucgaauaagauacagauacggaggaaggaucaacuuuaccuacucucacacaaucuucuucauauucucuucaauuuuuauuuuuuuauuguucguauuuuuuuucccagauuagauuucccuguuaaucaccaugguauuaaca
......................uggaucuuguuugGcagcacu...............................................................................................................................................................................  2      1     LT-1
......................uggaucuuguuuNacagcacu...............................................................................................................................................................................  1      1     LCK-1
......................uAgaucuuguuugacagcacu...............................................................................................................................................................................  30     1     LT-2
......................uggaucuuguuugacagcacu...............................................................................................................................................................................  4848   0     LCK-1
......................uggaucuCguuugacagcacu...............................................................................................................................................................................  4      1     JCK-3
......................uggaucuuguuuAacagcacu...............................................................................................................................................................................  1      1     LCK-3
......................uggaucuuguuNgacagcacu...............................................................................................................................................................................  1      1     JCK-2
......................uggaucCuguuugacagcacu...............................................................................................................................................................................  12     1     JCK-2
......................uggaucuuguuuAacagcacu...............................................................................................................................................................................  3      1     LT-1
......................uggaucuuguuugacGgcacu...............................................................................................................................................................................  3      1     LT-3
......................uggaucCuguuugacagcacu...............................................................................................................................................................................  1      1     LCK-1
......................uggaucuuguuAgacagcacu...............................................................................................................................................................................  1      1     JCK-2
......................uggaucuuUuuugacagcacu...............................................................................................................................................................................  3      1     LT-2
......................uUgaucuuguuugacagcacu...............................................................................................................................................................................  1      1     JT-3
......................uggaucCuguuugacagcacu...............................................................................................................................................................................  4      1     LT-1
......................uNgaucuuguuugacagcacu...............................................................................................................................................................................  4      1     JT-1
......................uggaucuuguuugacagcCcu...............................................................................................................................................................................  2      1     JT-1
......................uggaucuuguuugacagcaUu...............................................................................................................................................................................  36     1     LCK-1
......................uggaucuuguuuCacagcacu...............................................................................................................................................................................  1      1     JCK-2
......................uAgaucuuguuugacagcacu...............................................................................................................................................................................  13     1     LCK-3
......................uggaucGuguuugacagcacu...............................................................................................................................................................................  1      1     LT-1
......................uggaucuuguuugacGgcacu...............................................................................................................................................................................  7      1     LT-2
......................uggCucuuguuugacagcacu...............................................................................................................................................................................  92     1     JCK-1
......................uggGucuuguuugacagcacu...............................................................................................................................................................................  3      1     LT-3
......................Cggaucuuguuugacagcacu...............................................................................................................................................................................  1      1     JT-1
......................uggaucuuguuugacCgcacu...............................................................................................................................................................................  29     1     JCK-2
......................ugUaucuuguuugacagcacu...............................................................................................................................................................................  4      1     JCK-3
......................ugUaucuuguuugacagcacu...............................................................................................................................................................................  2      1     LT-2
......................uggaucuuguuugacagcCcu...............................................................................................................................................................................  74     1     JCK-2
......................uggaucuuNuuugacagcacu...............................................................................................................................................................................  1      1     JCK-3
......................uggaucuuguuuAacagcacu...............................................................................................................................................................................  1      1     LCK-1
......................uggaCcuuguuugacagcacu...............................................................................................................................................................................  3      1     LT-1
......................uggaucuuguuugacagcUcu...............................................................................................................................................................................  6      1     LT-2
......................uggaucuuguuugacagcaUu...............................................................................................................................................................................  1426   1     JCK-1
......................uNgaucuuguuugacagcacu...............................................................................................................................................................................  3      1     LT-1
......................uggaGcuuguuugacagcacu...............................................................................................................................................................................  2      1     JCK-3
......................uggaucuuguuuAacagcacu...............................................................................................................................................................................  1      1     LT-3
......................uggaucuuguuugaGagcacu...............................................................................................................................................................................  8      1     LCK-1
......................uggaucuuguuugGcagcacu...............................................................................................................................................................................  3      1     JCK-2
......................uggaucuuAuuugacagcacu...............................................................................................................................................................................  4      1     JCK-3
......................uggaucuuguuugacaCcacu...............................................................................................................................................................................  5      1     JCK-3
......................uggGucuuguuugacagcacu...............................................................................................................................................................................  1      1     JT-1
......................uAgaucuuguuugacagcacu...............................................................................................................................................................................  22     1     LT-3
......................uggaucuuguuugacCgcacu...............................................................................................................................................................................  28     1     JCK-3
......................uggaucuuguuGgacagcacu...............................................................................................................................................................................  10     1     JCK-1
......................uggaucuuguuugacagUacu...............................................................................................................................................................................  11     1     LT-2
......................uggaucuuguuugacagcacu...............................................................................................................................................................................  1709   0     JT-1
......................uggaucuuguuugacCgcacu...............................................................................................................................................................................  19     1     JCK-1
......................uggCucuuguuugacagcacu...............................................................................................................................................................................  152    1     JCK-2
......................uggaucuuguuugGcagcacu...............................................................................................................................................................................  5      1     LCK-1
......................uggaucuuguuugacaCcacu...............................................................................................................................................................................  3      1     JCK-2
......................uggaucuuguuugacagcCcu...............................................................................................................................................................................  29     1     LT-1
......................uggaucuuguuugacGgcacu...............................................................................................................................................................................  1      1     JT-3
......................uggaucuugCuugacagcacu...............................................................................................................................................................................  1      1     LT-3
......................uggaucuGguuugacagcacu...............................................................................................................................................................................  1      1     JCK-2
......................uggaucuuguuugacagcCcu...............................................................................................................................................................................  16     1     LCK-3
......................uAgaucuuguuugacagcacu...............................................................................................................................................................................  4      1     LCK-2
......................uggaucuuguuGgacagcacu...............................................................................................................................................................................  1      1     LT-3
......................uggaucuuguuuAacagcacu...............................................................................................................................................................................  1      1     JT-1
......................uggaucuuguuugUcagcacu...............................................................................................................................................................................  2      1     JCK-2
......................uggNucuuguuugacagcacu...............................................................................................................................................................................  22     1     JCK-1
......................uggaucuuguuugaGagcacu...............................................................................................................................................................................  1      1     JT-1
......................uggaucuuguCugacagcacu...............................................................................................................................................................................  7      1     JCK-3
......................uggaucuuguuugacaCcacu...............................................................................................................................................................................  1      1     LT-3
......................uggaucuuguCugacagcacu...............................................................................................................................................................................  1      1     LCK-2
......................uggaucuuguuugacagcacu...............................................................................................................................................................................  20263  0     LCK-3
......................uggaucuuguuuAacagcacu...............................................................................................................................................................................  9      1     JCK-2
......................Gggaucuuguuugacagcacu...............................................................................................................................................................................  1      1     JCK-2
......................uggCucuuguuugacagcacu...............................................................................................................................................................................  9      1     LCK-2
......................uggCucuuguuugacagcacu...............................................................................................................................................................................  65     1     LT-1
```

Mature Star

uccaucuuguucguuuacccucuggaucuuguuugacagcacucucuccuaucuuguuugacugaugaugauggagugcugucgacaagaucuagaagguacugucgaauaagauacagauacggaggaaggaucaacuuuaccuacucucacacaaucuucuucauauucucuucaauuuuuauuuuuuuauuguucguauuuuuuuucccagauuagauuucccuguuaaucaccaugguauuaaca

......................uggaucuuguGugacagcacu	15	1	JCK-1
......................uggaucuuguuugacGgcacu	2	1	LCK-3
......................uggaucuuguuugacagcNcu	1	1	LCK-1
......................ugUaucuuguuugacagcacu	1	1	LCK-2
......................uggaCcuuguuugacagcacu	1	1	LCK-3
......................uggaucuuguuugCcagcacu	80	1	LT-2
......................uggaucuuguuugacagcacu	48441	0	JCK-3
......................uggaucuuguCugacagcacu	6	1	JCK-2
......................uggCucuuguuugacagcacu	200	1	LT-2
......................uggaucuuguuuUacagcacu	2	1	JCK-3
......................Cggaucuuguuugacagcacu	1	1	JT-3
......................uggaucuuguuGgacagcacu	1	1	JCK-2
......................uggaucuuguuugacaCcacu	5	1	JCK-1
......................uggaucuuguuugacagcGcu	1	1	JT-3
......................uggaucuuguuugacagUacu	1	1	JT-2
......................uggaucuuguuugacagcacG	3	1	LT-2
......................uggaucuuguuugacagcaUu	55	1	JT-1
......................uggaucuuguuugCcagcacu	105	1	JCK-3
......................uggaucuugCuugacagcacu	2	1	LT-1
......................ugAaucuuguuugacagcacu	10	1	LT-2
......................uggaucuuAuuugacagcacu	6	1	LT-1
......................uggaucuuguuugaAagcacu	5	1	JCK-3
......................uggaucuuguuugacagcaAu	1	1	LT-1
......................uggaucuuguuugacagcaUu	21	1	JT-3
......................uNgaucuuguuugacagcacu	66	1	JCK-1
......................uggaucuuguuugacagcacu	979	0	JT-3
......................uggNucuuguuugacagcacu	1	1	JCK-2
......................uggaucuuguuugaAagcacu	4	1	JCK-2
......................uggaucuCguuugacagcacu	3	1	LCK-3
......................uggaCcuuguuugacagcacu	1	1	LCK-2
......................Cggaucuuguuugacagcacu	4	1	LT-2
......................uggaucuuUuuugacagcacu	1	1	LCK-3
......................uUgaucuuguuugacagcacu	2	1	LCK-3
......................uggaucuuguuugacGgcacu	9	1	JCK-3
......................ugAaucuuguuugacagcacu	8	1	JCK-3
......................uggCucuuguuugacagcacu	95	1	LT-3
......................uggaucuuguuuUacagcacu	6	1	JCK-2
......................uggauUuuguuugacagcacu	11	1	JCK-2
......................uggaucuuguuugacagcaGu	2	1	JCK-1
......................uggaucuuUuuugacagcacu	4	1	JCK-2
......................uggaucuuguuugacagcCcu	53	1	JCK-1
......................uggaucuuguuugCcagcacu	1	1	JT-3
......................uggaucuuguuugacCgcacu	11	1	LT-3
......................uggaucuuguuugacagcacu	5029	0	LCK-2
......................uggaucuuguuugacagAacu	3	1	LT-1
......................uggaucuuCuuugacagcacu	1	1	JCK-1
......................uggaucuuguuuUacagcacu	6	1	JCK-1
......................uggaCcuuguuugacagcacu	14	1	JCK-2
......................uggaucuuguuugCcagcacu	5	1	JT-1
......................uggaucuuguuugacagcacG	10	1	JCK-1
......................uggaucuuguuuAacagcacu	7	1	JCK-3
......................uggaucuuguuugacagcaAu	3	1	JCK-3
......................uggaucuugCuugacagcacu	2	1	JCK-1
......................uggaucGuguuugacagcacu	2	1	JCK-1
......................uggaucuuguuugacagcaAu	1	1	LCK-3
......................uggaucuuAuuugacagcacu	6	1	JCK-1
......................uggaucuuguuugacagcaUu	102	1	LCK-3
......................uggaucuuguuuCacagcacu	1	1	JCK-3
......................uggaucuuguuugGcagcacu	3	1	JCK-1
......................uAgaucuuguuugacagcacu	14	1	LT-1
......................uggaucuCguuugacagcacu	3	1	LT-3
......................uggaucuuguuugacagcaAu	1	1	LCK-2
......................uggaucuuguuugacCgcacu	7	1	LCK-3
......................uggaucuuguCugacagcacu	6	1	JCK-1
......................uggaucuuguuugCcagcacu	2	1	JT-2
......................uggaucuuCuuugacagcacu	1	1	JCK-3
......................uggaucuuguuugacagcaUu	37	1	LCK-2
......................uNgaucuuguuugacagcacu	1	1	LCK-1
......................uggaucuuguuugacCgcacu	3	1	JT-1
......................uggaucuuguCugacagcacu	1	1	LT-3

Mature **Star**

uccaucuuguucguuuacccuc**uggaucuuguuugacagcacu**cucuccuaucuuguuugacugaugaugauggga**gugcugucgacaagaucuaga**agguacugucgaauaagauacagauacggaggaaggaucaacuuuaccuacucucacacaaucuucuucauauucucuucaauuuuuauuuuuuuuauuguucguauuuuuuuucccagauuagauuucccuguuaaucaccaugguauuaaca

......................uggaucuuAuuugacagcacu......	3	1	LT-3
......................uggaucuuguuugacagcacG......	16	1	JCK-2
......................uggaucuuguuugacaCcacu......	1	1	JT-1
......................uggaucuuguuugacagcGcu......	2	1	JCK-2
......................uUgaucuuguuugacagcacu......	2	1	LT-1
......................uggaucuuAuuugacagcacu......	1	1	LCK-1
......................uggaucuuguuugacagcUcu......	1	1	LT-3
......................uggGucuuguuugacagcacu......	5	1	JCK-3
......................uggaucuuUuuugacagcacu......	6	1	JCK-3
......................uggaucuuguuugacagNacu......	3	1	JCK-3
......................uggaucuCguuugacagcacu......	1	1	LCK-1
......................uggaucuuguuugacagcacu......	38814	0	JCK-1
......................uggNucuuguuugacagcacu......	3	1	JCK-3
......................uggaucuuguuugacagcCcu......	31	1	LT-3
......................uggaucuuguuuCacagcacu......	2	1	JCK-1
......................Cggaucuuguuugacagcacu......	7	1	JCK-3
......................ugNaucuuguuugacagcacu......	1	1	LCK-1
......................uggauUuuguuugacagcacu......	1	1	LCK-3
......................uggaucCuguuugacagcacu......	8	1	JCK-3
......................uggaucuuguCugacagcacu......	5	1	JT-2
......................uggaucuuAuuugacagcacu......	1	1	LCK-2
......................uggauUuuguuugacagcacu......	2	1	LT-3
......................uCgaucuuguuugacagcacu......	1	1	JCK-3
......................ugAaucuuguuugacagcacu......	6	1	LT-1
......................uUgaucuuguuugacagcacu......	4	1	JCK-2
......................ugCaucuuguuugacagcacu......	1	1	JCK-3
......................uggaucuuguuuAacagcacu......	2	1	LT-2
......................uAgaucuuguuugacagcacu......	5	1	JCK-1
......................ugUaucuuguuugacagcacu......	1	1	JT-1
......................uggaucuuguuugaAagcacu......	3	1	LT-1
......................uggaucuuguuugacagcacu......	20839	0	LT-2
......................Cggaucuuguuugacagcacu......	3	1	LT-1
......................uggCucuuguuugacagcacu......	2	1	JT-2
......................uggaucuuguuugacagcacG......	13	1	JCK-3
......................uggaucuuguuuGcagcacu......	3	1	JCK-3
......................uggaucuCguuugacagcacu......	10	1	JCK-2
......................uggaucuuguuugacagcaAu......	3	1	JCK-1
......................uggaucuuguuugCcagcacu......	67	1	LT-3
......................uCgaucuuguuugacagcacu......	1	1	LCK-3
......................uggauAuuguuugacagcacu......	1	1	JCK-3
......................uggCucuuguuugacagcacu......	8	1	LCK-1
......................uggaucuuguuugacagGacu......	24	1	JCK-1
......................ugCaucuuguuugacagcacu......	1	1	LCK-3
......................uAgaucuuguuugacagcacu......	15	1	JCK-3
......................ugAaucuuguuugacagcacu......	3	1	LCK-2
......................uggaucuuguuugCcagcacu......	36	1	LCK-3
......................uNgaucuuguuugacagcacu......	55	1	JCK-3
......................uggaucuuguuugCcagcacu......	159	1	JCK-2
......................uggaucuuguuugacagcUcu......	5	1	LT-1
......................ugAaucuuguuugacagcacu......	11	1	LT-3
......................uggaucuuguuugacagUacu......	18	1	JCK-1
......................uggaucuuguuAgacagcacu......	1	1	LCK-3
......................uggaucuuUuuugacagcacu......	3	1	JCK-1
......................uggaucuugGuugacagcacu......	1	1	JCK-3
......................ugNaucuuguuugacagcacu......	65	1	LT-2
......................uggaucuuguuugacagGacu......	2	1	JCK-2
......................uggaucuuguuugacagcGcu......	2	1	JCK-1
......................uggCucuuguuugacagcacu......	1	1	JT-3
......................uggaucuugCuugacagcacu......	6	1	JCK-3
......................uggaucuuguuugacagcaNu......	42	1	JCK-1
......................uggaucuuAuuugacagcacu......	4	1	LCK-3
......................uggaucuuguuugacagcCcu......	17	1	JCK-3
......................uggaucuuguuugacagcaAu......	4	1	JT-3
......................uggauGuuguuugacagcacu......	1	1	JCK-3
......................uNgaucuuguuugacagcacu......	1	1	LCK-2
......................uggaucuuguuugCcagcacu......	7	1	LCK-2
......................uggaucuuguuugacagcacu......	27651	0	LT-3
......................uggaucuuguuugacGgcacu......	10	1	JCK-2
......................uUgaucuuguuugacagcacu......	10	1	JCK-3
......................uggaCcuuguuugacagcacu......	4	1	JCK-1

```
                      Mature                                                      Star
uccaucuuguucguuuacccucuggaucuuguuugacagcacucucuccuaucuuguuugacugaugaugaugggagugcugucgacaagaucuagaagguacugucgaauaagauacagauacggaggaaggaucaacuuuaccuacucucacacaaucuucuucauauucucuucaauuuuuauuuuuuuauuguucguauuuuuuuucccagauuagauuucccuguuaaucaccaugguauuaaca
......................uggaucuuguuugacagcacu...............................................................................................................................................................................................................   66730   0   JCK-2
......................uggaucuuguuugacagcNcu...............................................................................................................................................................................................................   22      1   JCK-1
......................Cggaucuuguuugacagcacu...............................................................................................................................................................................................................   9       1   LT-3
......................ugAaucuuguuugacagcacuc..............................................................................................................................................................................................................   2       1   LT-3
......................uggaucuuguuugacagNacuc..............................................................................................................................................................................................................   1       1   JCK-3
......................uggaucuuguuugCcagcacuc..............................................................................................................................................................................................................   2       1   LT-2
......................uggaucuuguuugacagcacuA..............................................................................................................................................................................................................   32      1   LT-1
......................uggaucuuguuugCcagcacuc..............................................................................................................................................................................................................   2       1   JT-1
......................uggaucuuguuugacCgcacuc..............................................................................................................................................................................................................   3       1   JCK-3
......................uggaucuuguuugacagcacuG..............................................................................................................................................................................................................   4       1   JCK-2
......................uggaucuuguuugacagcacuA..............................................................................................................................................................................................................   11      1   JT-1
......................uggaucuuguuugacagcaUuc..............................................................................................................................................................................................................   7       1   JCK-1
......................uggaucuuguuugacagcacuc..............................................................................................................................................................................................................   627     0   LT-2
......................uggaucuuguGugacagcacuc..............................................................................................................................................................................................................   5       1   JCK-1
......................uggaucuuguuugacagcacGc..............................................................................................................................................................................................................   1       1   JCK-3
......................uggaucuuguuugacGgcacuc..............................................................................................................................................................................................................   1       1   LT-1
......................uggaucuCguuugacagcacuc..............................................................................................................................................................................................................   3       1   JCK-2
......................uggaucuuguuugacagcacuU..............................................................................................................................................................................................................   27      1   LT-2
......................uggaucuuguuugacagcacuc..............................................................................................................................................................................................................   76      0   LCK-1
......................uggaucuuguuugacagcacuA..............................................................................................................................................................................................................   33      1   LT-2
......................uggaucCuguuugacagcacuc..............................................................................................................................................................................................................   5       1   JCK-3
......................uggaucuuAuuugacagcacuc..............................................................................................................................................................................................................   4       1   JCK-3
......................uggGucuuguuugacagcacuc..............................................................................................................................................................................................................   1       1   JCK-3
......................uAgaucuuguuugacagcacuc..............................................................................................................................................................................................................   1       1   LT-2
......................uggaucuuguuugacagcacuU..............................................................................................................................................................................................................   68      1   JCK-1
......................uggaucCuguuugacagcacuc..............................................................................................................................................................................................................   1       1   JCK-1
......................uggaucuuguuugacagcacuU..............................................................................................................................................................................................................   48      1   LT-3
......................uggaucuugGuugacagcacuc..............................................................................................................................................................................................................   3       1   JCK-1
......................uggaucuuguuNgacagcacuc..............................................................................................................................................................................................................   29      1   JCK-1
......................uggaucuuguuugacagcaUuc..............................................................................................................................................................................................................   8       1   JCK-3
......................uggGucuuguuugacagcacuc..............................................................................................................................................................................................................   4       1   JCK-2
......................uggCucuuguuugacagcacuc..............................................................................................................................................................................................................   36      1   JCK-1
......................uggaucuuguuugacagcacuU..............................................................................................................................................................................................................   1       1   JT-2
......................uggaucuuguuugacagcUcuc..............................................................................................................................................................................................................   2       1   JCK-2
......................uggaucuuguuugacagcacuG..............................................................................................................................................................................................................   1       1   LT-2
......................uggaucuuguuugacagcacuc..............................................................................................................................................................................................................   520     0   LCK-3
......................ugAaucuuguuugacagcacuc..............................................................................................................................................................................................................   1       1   LT-1
......................uggaucuuguuugacCgcacuc..............................................................................................................................................................................................................   1       1   LT-2
......................uggaucuuguuugCcagcacuc..............................................................................................................................................................................................................   44      1   JCK-2
......................uggaucuuguuugCcagcacuc..............................................................................................................................................................................................................   19      1   JCK-3
......................uggaucuuguuugacagcacuG..............................................................................................................................................................................................................   3       1   LT-3
......................ugNaucuuguuugacagcacuc..............................................................................................................................................................................................................   2       1   LT-2
......................uggaucuuguuugacagcacuU..............................................................................................................................................................................................................   18      1   LCK-1
......................ugAaucuuguuugacagcacuc..............................................................................................................................................................................................................   1       1   LT-2
......................uAgaucuuguuugacagcacuc..............................................................................................................................................................................................................   7       1   JCK-2
......................ugCaucuuguuugacagcacuc..............................................................................................................................................................................................................   1       1   JCK-2
......................uggaucuuguuugacCgcacuc..............................................................................................................................................................................................................   5       1   JCK-1
......................uggaucuuguuuAacagcacuc..............................................................................................................................................................................................................   3       1   JCK-2
......................uggaucuuguuugacagcacuc..............................................................................................................................................................................................................   1262    0   LT-1
......................uggaucuuguuugacagcacuN..............................................................................................................................................................................................................   9       1   JCK-1
......................uggaucuugGuugacagcacuc..............................................................................................................................................................................................................   3       1   JCK-2
......................uggaucuuguuugacCgcacuc..............................................................................................................................................................................................................   1       1   LT-1
......................uggaucuuguuugacagcCcuc..............................................................................................................................................................................................................   7       1   JCK-1
......................uggaucuuguuuAacagcacuc..............................................................................................................................................................................................................   1       1   JCK-1
......................uggaucuuguuugCcagcacuc..............................................................................................................................................................................................................   2       1   LT-3
......................uggaucuuguuugacagcacuc..............................................................................................................................................................................................................   9941    0   JCK-1
......................uNgaucuuguuugacagcacuc..............................................................................................................................................................................................................   17      1   JCK-2
......................uggaucuuguuugacagcacuU..............................................................................................................................................................................................................   64      1   LCK-3
......................uAgaucuuguuugacagcacuc..............................................................................................................................................................................................................   10      1   JCK-3
......................uggaucuuguuugGcagcacuc..............................................................................................................................................................................................................   3       1   JCK-2
......................uggaucuuguuugacagcacuG..............................................................................................................................................................................................................   7       1   LT-1
......................uggaucuuguuugacagcacuA..............................................................................................................................................................................................................   41      1   JCK-3
......................uUgaucuuguuugacagcacuc..............................................................................................................................................................................................................   1       1   JCK-3
......................uggaCcuuguuugacagcacuc..............................................................................................................................................................................................................   1       1   LT-2
......................uggaucuCguuugacagcacuc..............................................................................................................................................................................................................   2       1   JCK-3
......................uggaucuuguuugacaCcacuc..............................................................................................................................................................................................................   1       1   JCK-3
......................uggaucuuguuugacagcUcuc..............................................................................................................................................................................................................   1       1   JCK-1
......................uggauUuuguuugacagcacuc..............................................................................................................................................................................................................   1       1   JCK-3
......................uNgaucuuguuugacagcacuc..............................................................................................................................................................................................................   15      1   JCK-3
......................uggaucuuguuugacagcacuc..............................................................................................................................................................................................................   1069    0   LT-3
```

```
                      Mature                                                      Star
uccaucuuguucguuuacccucuggaucuuguuugacagcacucucuccuaucuuguuugacugaugaugaugggagugcugucgacaagaucuagaagguacugucgaauaagauacagauacggaggaaggaucaacuuuaccuacucucacacaaucuucuucauauucucuucaauuuuuauuuuuuuauuguucguauuuuuuuucccagauuagauuucccuguuaaucaccaugguauuaaca
......................uggaucuuNuuugacagcacuc..............................................................................................................................................................................................................   1     1   JCK-3
......................uUgaucuuguuugacagcacuc..............................................................................................................................................................................................................   1     1   LT-2
......................uggCucuuguuugacagcacuc..............................................................................................................................................................................................................   2     1   LCK-1
......................uggaucuuguuugacagcacuA..............................................................................................................................................................................................................   38    1   JCK-1
......................uggaucuuguuugacagcaUuc..............................................................................................................................................................................................................   3     1   LT-2
......................uggaucuuguuugNcagcacuc..............................................................................................................................................................................................................   1     1   LCK-1
......................uggaucuuguuugacCgcacuc..............................................................................................................................................................................................................   13    1   JCK-2
......................uggCucuuguuugacagcacuc..............................................................................................................................................................................................................   3     1   LCK-3
......................uggaucuuguuugacagcGcuc..............................................................................................................................................................................................................   3     1   JCK-2
......................uggaucuuguuugacagUacuc..............................................................................................................................................................................................................   5     1   JCK-2
......................uggaucuuguuugacagcacuU..............................................................................................................................................................................................................   4     1   JT-3
......................uggaucuugCuugacagcacuc..............................................................................................................................................................................................................   1     1   JCK-2
......................uggaucuuguuugacagcaAuc..............................................................................................................................................................................................................   1     1   JCK-2
......................uggaucuuAuuugacagcacuc..............................................................................................................................................................................................................   1     1   LT-2
......................Cggaucuuguuugacagcacuc..............................................................................................................................................................................................................   5     1   JCK-3
......................uggaucuuguuugacaAcacuc..............................................................................................................................................................................................................   3     1   JCK-2
......................uggaucuuguuugaGagcacuc..............................................................................................................................................................................................................   4     1   JCK-2
......................uggaucuuguuugacagcNcuc..............................................................................................................................................................................................................   7     1   JCK-1
......................uggaucuuguuuAacagcacuc..............................................................................................................................................................................................................   1     1   JCK-3
......................uggaucuuguuugacagGacuc..............................................................................................................................................................................................................   7     1   JCK-1
......................uggaucuugCuugacagcacuc..............................................................................................................................................................................................................   1     1   JCK-3
......................uggaucuuguuugacagcacuc..............................................................................................................................................................................................................   220   0   JT-1
......................uggaucuuguuugCcagcacuc..............................................................................................................................................................................................................   24    1   JCK-1
......................uggaucuuguuugCcagcacuc..............................................................................................................................................................................................................   1     1   JT-2
......................uggaucuuguuugacCgcacuc..............................................................................................................................................................................................................   2     1   LCK-3
......................uggNucuuguuugacagcacuc..............................................................................................................................................................................................................   7     1   JCK-1
......................uggCucuuguuugacagcacuc..............................................................................................................................................................................................................   33    1   JCK-3
......................uggaucuugGuugacagcacuc..............................................................................................................................................................................................................   3     1   JCK-3
......................uggaucuuguuugacagcacNc..............................................................................................................................................................................................................   3     1   JCK-1
......................uggaucuuguuGgacagcacuc..............................................................................................................................................................................................................   3     1   JCK-1
......................ugAaucuuguuugacagcacuc..............................................................................................................................................................................................................   2     1   JCK-1
......................uggaucuuguuugacagcaNuc..............................................................................................................................................................................................................   11    1   JCK-1
......................uggaucuuguuugCcagcacuc..............................................................................................................................................................................................................   7     1   LT-1
......................uggaucuuguuugacagcacuA..............................................................................................................................................................................................................   34    1   LT-3
......................uggaucuuguuugacagcCcuc..............................................................................................................................................................................................................   9     1   JCK-3
......................uggaucuuguuugacaAcacuc..............................................................................................................................................................................................................   4     1   JCK-1
......................uggaucuuguuugacagcCcuc..............................................................................................................................................................................................................   7     1   JCK-2
......................uggaucuuAuuugacagcacuc..............................................................................................................................................................................................................   4     1   JCK-2
......................uAgaucuuguuugacagcacuc..............................................................................................................................................................................................................   1     1   LCK-3
......................Cggaucuuguuugacagcacuc..............................................................................................................................................................................................................   6     1   JCK-2
......................uggaucuuguuugacagcGcuc..............................................................................................................................................................................................................   1     1   JCK-1
......................uggaucuuguCugacagcacuc..............................................................................................................................................................................................................   1     1   JCK-1
......................uggaucuuguuugacagcacuc..............................................................................................................................................................................................................   152   0   JT-3
......................uggaucuuguuugGcagcacuc..............................................................................................................................................................................................................   1     1   JCK-1
......................Cggaucuuguuugacagcacuc..............................................................................................................................................................................................................   1     1   LCK-3
......................uggaucuuguuugacagcacuc..............................................................................................................................................................................................................   70    0   LCK-2
......................uggaucuuguuugacagcCcuc..............................................................................................................................................................................................................   1     1   JT-3
......................uggaucuuguuugacagcacuU..............................................................................................................................................................................................................   97    1   JCK-2
......................uggaucuuguuugacagcacuG..............................................................................................................................................................................................................   11    1   JCK-1
......................uAgaucuuguuugacagcacuc..............................................................................................................................................................................................................   3     1   JCK-1
......................uggaucuuguuugacGgcacuc..............................................................................................................................................................................................................   2     1   JCK-3
......................uggaucuuguuugacagcacuN..............................................................................................................................................................................................................   4     1   LT-2
......................uggaucuuUuuugacagcacuc..............................................................................................................................................................................................................   1     1   JCK-3
......................ugUaucuuguuugacagcacuc..............................................................................................................................................................................................................   3     1   JCK-3
......................ugUaucuuguuugacagcacuc..............................................................................................................................................................................................................   1     1   JCK-2
......................uggGucuuguuugacagcacuc..............................................................................................................................................................................................................   1     1   JCK-1
......................uAgaucuuguuugacagcacuc..............................................................................................................................................................................................................   1     1   LT-3
......................uUgaucuuguuugacagcacuc..............................................................................................................................................................................................................   1     1   JCK-2
......................uggaucuuguuugCcagcacuc..............................................................................................................................................................................................................   3     1   LCK-3
......................uggCucuuguuugacagcacuc..............................................................................................................................................................................................................   7     1   LT-3
......................uggCucuuguuugacagcacuc..............................................................................................................................................................................................................   1     1   JT-1
......................uggaCcuuguuugacagcacuc..............................................................................................................................................................................................................   9     1   JCK-3
......................uggaucuCguuugacagcacuc..............................................................................................................................................................................................................   2     1   JCK-1
......................uggaucuuguuugacaAcacuc..............................................................................................................................................................................................................   6     1   JCK-3
......................uggaucuuguuuUacagcacuc..............................................................................................................................................................................................................   1     1   JCK-3
......................uggaucuuUuuugacagcacuc..............................................................................................................................................................................................................   2     1   JCK-2
......................ugAaucuuguuugacagcacuc..............................................................................................................................................................................................................   4     1   JCK-2
......................uggaucuuguuugacagcCcuc..............................................................................................................................................................................................................   1     1   LCK-1
......................uggauAuuguuugacagcacuc..............................................................................................................................................................................................................   1     1   JCK-1
......................uggCucuuguuugacagcacuc..............................................................................................................................................................................................................   4     1   LT-2
```

```
                      Mature                                              Star
uccaucuuguucguuuacccucuggaucuuguuugacagcacucucuccuaucuuguuugacugaugaugaugggagugcugucgacaagaucuagaagguacugucgaauaagauacagauacggaggaaggaucaacuuuaccuacucucacacaaucuucuucauauucucuucaauuuuuauuuuuuuauuguucguauuuuuuuucccagauuagauuucccuguuaaucaccaugguauuaaca
......................uggaucuuguuugacagcaUuc..............................................................................................................................................................................................    5      1    JCK-2
......................uUgaucuuguuugacagcacuc..............................................................................................................................................................................................    1      1    JCK-1
......................uggaGcuuguuugacagcacuc..............................................................................................................................................................................................    1      1    JCK-3
......................uggaucuuguuugacagcacuA..............................................................................................................................................................................................    12     1    LCK-3
......................uggCucuuguuugacagcacuc..............................................................................................................................................................................................    36     1    JCK-2
......................uggaucuuguuugacagcacuU..............................................................................................................................................................................................    22     1    LCK-2
......................Cggaucuuguuugacagcacuc..............................................................................................................................................................................................    2      1    JCK-1
......................uggaucuuguuugacagcacuc..............................................................................................................................................................................................    17179  0    JCK-2
......................uNgaucuuguuugacagcacuc..............................................................................................................................................................................................    26     1    JCK-1
......................ugAaucuuguuugacagcacuc..............................................................................................................................................................................................    2      1    JCK-3
......................uggaucuuguuugacagcacGc..............................................................................................................................................................................................    1      1    JCK-2
......................uAgaucuuguuugacagcacuc..............................................................................................................................................................................................    2      1    LT-1
......................Cggaucuuguuugacagcacuc..............................................................................................................................................................................................    1      1    LT-2
......................uggCucuuguuugacagcacuc..............................................................................................................................................................................................    4      1    LT-1
......................uggaucuuguuugacagcacuc..............................................................................................................................................................................................    12872  0    JCK-3
......................uggaucuuguuuUacagcacuc..............................................................................................................................................................................................    1      1    JCK-1
......................uggaCcuuguuugacagcacuc..............................................................................................................................................................................................    6      1    JCK-1
......................uggaucuuguuugacagcacuA..............................................................................................................................................................................................    59     1    JCK-2
......................uggaucuuguuugacagcacuU..............................................................................................................................................................................................    47     1    LT-1
......................Cggaucuuguuugacagcacuc..............................................................................................................................................................................................    1      1    LT-1
......................uggaucuuguuugacagcacuc..............................................................................................................................................................................................    138    0    JT-2
......................ugUaucuuguuugacagcacuc..............................................................................................................................................................................................    1      1    JCK-1
......................uggauUuuguuugacagcacuc..............................................................................................................................................................................................    5      1    JCK-1
......................uggaucuuguuugacagcacGc..............................................................................................................................................................................................    1      1    JCK-1
......................uggauUuuguuugacagcacuc..............................................................................................................................................................................................    7      1    JCK-2
......................uggaucuuguuugacagcacuU..............................................................................................................................................................................................    85     1    JCK-3
......................uggaucCuguuugacagcacuc..............................................................................................................................................................................................    2      1    JCK-2
......................uggaucuuguuugacagcacuG..............................................................................................................................................................................................    2      1    JCK-3
......................uggCucuuguuugacagcacuc..............................................................................................................................................................................................    1      1    JT-2
......................uggaucuuguuugaAagcacuc..............................................................................................................................................................................................    4      1    JCK-2
......................uggaucuuguuugacagUacuc..............................................................................................................................................................................................    1      1    JCK-1
......................uggaucuuguuugacagcacucu.............................................................................................................................................................................................    80     0    JCK-3
......................uggaucuuguuugacagcacuUu.............................................................................................................................................................................................    75     1    LCK-3
......................uggaucuuguuugacagcacuUu.............................................................................................................................................................................................    21     1    JT-2
......................uggaucuuguuugacagcacuAu.............................................................................................................................................................................................    3      1    JCK-1
......................uggaucuuguuugacagcacuUu.............................................................................................................................................................................................    21     1    JT-3
......................uggCucuuguuugacagcacucu.............................................................................................................................................................................................    1      1    LT-2
......................uggaucuuguuugacagcacucA.............................................................................................................................................................................................    14     1    JT-3
......................uggaucuuguuugacagcacuUu.............................................................................................................................................................................................    53     1    LT-3
......................uggaucuuguuugacagcacucA.............................................................................................................................................................................................    23     1    LT-1
......................uggaucuuguuugacagcacucA.............................................................................................................................................................................................    79     1    JCK-1
......................uggaucuuguuugacagcacucG.............................................................................................................................................................................................    4      1    JCK-3
......................uggaucuuguuugacagcacucA.............................................................................................................................................................................................    140    1    JCK-2
......................uggaucuuguuNgacagcacucu.............................................................................................................................................................................................    3      1    JCK-1
......................uggaucuuguuugacagcacuUu.............................................................................................................................................................................................    62     1    LT-1
......................uggaucuuguuugacagcacucu.............................................................................................................................................................................................    14     0    LT-2
......................uggaucuuguuugacagcacucu.............................................................................................................................................................................................    26     0    LT-1
......................uggaucuuguuugacagcacuGu.............................................................................................................................................................................................    5      1    LT-1
......................uggaucuuguuugacagcacucu.............................................................................................................................................................................................    111    0    JCK-2
......................uggaucuuguuugacagcacuAu.............................................................................................................................................................................................    2      1    JCK-2
......................uggaucuuguuugacagcacucG.............................................................................................................................................................................................    5      1    JCK-2
......................uggaucuuguuugacagcacuUu.............................................................................................................................................................................................    19     1    LCK-2
......................uggaucuuguuugacagcacucA.............................................................................................................................................................................................    100    1    JCK-3
......................uggaucuuguuugacagcacuGu.............................................................................................................................................................................................    1      1    LT-3
......................uggaucuuguuugacagcacuUu.............................................................................................................................................................................................    109    1    JCK-1
......................uggaucuuguuugacagcacuGu.............................................................................................................................................................................................    2      1    LT-2
......................uggaucuuguuugacagcacuAu.............................................................................................................................................................................................    1      1    JCK-3
......................uggaucuuguuugacagcacucA.............................................................................................................................................................................................    14     1    LT-2
......................uggaucuuguuugacagcacuUu.............................................................................................................................................................................................    39     1    JT-1
......................uggaucuuguuugacagcacucA.............................................................................................................................................................................................    6      1    LT-3
......................uggaucuuguuugacagcacucA.............................................................................................................................................................................................    24     1    LCK-3
......................uggaucuuguuugacagcacucu.............................................................................................................................................................................................    16     0    LCK-3
......................uggaucuuguuugacagcacuUu.............................................................................................................................................................................................    71     1    LT-2
......................uggaucuuguuugacagcacuUu.............................................................................................................................................................................................    159    1    JCK-2
......................uggaucuuguuugacagcacucu.............................................................................................................................................................................................    10     0    LT-3
......................uggaucuuguuugacagcacuGu.............................................................................................................................................................................................    6      1    JCK-2
......................uggaucuuguuugacagcacucG.............................................................................................................................................................................................    3      1    JCK-1
......................uggaucuuguuugacagcacucu.............................................................................................................................................................................................    90     0    JCK-1
......................uggaucuuguuugacagcacuUu.............................................................................................................................................................................................    23     1    LCK-1
......................uggaucuuguuugacagcacuUu.............................................................................................................................................................................................    112    1    JCK-3
```

Mature **Star**

```
uccaucuuguucguuuacccucuggaucuuguuugacagcacucucuccuaucuuguuugacugaugaugaugggagugcugucgacaagaucuagaagguacugucgaauaagauacagauacggaggaaggaucaacuuuaccuacucucacacaaucuucuucauauucucuucaauuuuuauuuuuuuauuguucguauuuuuuuucccagauuagauuucccuguuaaucaccaugguauuaaca
......................uggaucuuguuugacagcacucA..........    10    1    JT-2
......................uggaucuuguuugacagcacuGu..........    1     1    JCK-1
......................uggaucuuguuugacagcacucAc.........    7     1    LT-1
......................uggaucuuguuugacagcacucuc.........    19    0    JCK-2
......................uggaucuuguuugacagcacucuc.........    3     0    LCK-3
......................uggaucuuguuugacagcacuGuc.........    1     1    LT-3
......................uggaucuuguuugacagcacucuA.........    2     1    JCK-1
......................uggaucuuguuugacagcacucuc.........    1     0    LT-1
......................uggaucuuguuugacagcacucuU.........    6     1    JCK-2
......................uggaucuuguuugacagcacucuc.........    12    0    JCK-1
......................uggaucuuguuugacagcacucuA.........    5     1    LT-2
......................uggaucuuguuugacagcacucuA.........    1     1    LT-3
......................uggaucuuguuugacagcacucAc.........    13    1    LCK-3
......................uggaucuuguuugacagcacucuU.........    7     1    LT-2
......................uggaucuuguuNgacagcacucuc.........    1     1    JCK-1
......................uggaucuuguuugacagcacucuc.........    1     0    LT-3
......................uggaucuuguuugacagcacucuc.........    19    0    JCK-3
......................uggaucuuguuugacagcacucuA.........    2     1    JCK-2
......................uggaucuuguuugacagcacucAc.........    2     1    LT-3
......................uggaucuuguuugacagcacucuA.........    2     1    JCK-3
......................uggaucuuguuugacagcacuUuc.........    3     1    JCK-2
......................uggaucuuguuugacagcacucuU.........    1     1    JCK-1
......................uggaucuuguuugacagcacucuU.........    9     1    LT-1
......................uggaucuuguuugacagcacucuA.........    1     1    LT-1
......................uggaucuuguuugacagcacucuAu........    1     1    LT-1
......................uggaucuuguuugacagcacucucu........    1     0    LT-3
......................uggaucuuguuugacagcacucuUu........    1     1    LT-1
......................uggaucuuguuugacagcacucuUu........    1     1    JCK-1
......................uggaucuuguuugacagcacucAcu........    1     1    JCK-2
......................uggaucuuguuugacagcacucucu........    2     0    JCK-3
......................uggaucuuguuugacagcacucucu........    1     0    JCK-1
......................uggaucuuguuugacagcacucuUu........    2     1    JCK-2
......................uggaucuuguuugacagcacucuUu........    6     1    LT-2
......................uggaucuuguuugacagcacucucu........    6     0    JCK-2
......................uggaucuuguuugacagcacucucGc.......    1     1    LT-1
......................uggaucuuguuugacagcacucuGuc.......    2     1    JCK-2
......................uggaucuuguuugacagcacucuGuc.......    1     1    JCK-3
......................uggaucuuguuugacagcacucucucc......    3     0    LCK-3
.......................ggaucuuguuugacagca..............    1     0    LT-1
.......................ggaucuuguuugacagca..............    1     0    JCK-1
.......................ggaucuuguuugacagca..............    2     0    JCK-3
.......................ggaucuuguuugacagcac.............    2     0    LT-1
.......................ggaucuuguuugacagcac.............    4     0    LCK-3
.......................ggaucuuguuugacagcac.............    1     0    LT-2
.......................ggaucuuguuugacagcacu............    3     0    JCK-2
.......................ggaucuuguuugacagcacu............    5     0    LT-1
.......................ggaucuuguuugacagcacu............    2     0    LCK-3
.......................ggaucuuguuugacagcacu............    5     0    LT-2
.......................ggaucuuguuugacagcacu............    4     0    JCK-3
.......................ggaucuuguuugacagcacu............    5     0    JCK-1
.......................ggaucuuguuugacagcacu............    2     0    LT-3
.......................ggaucuuguuugacagcacuc...........    6     0    JCK-2
.......................ggaucuuguuugacagcacuc...........    6     0    JCK-1
.......................ggaucuuguuugacagcacuc...........    14    0    JCK-3
.......................ggaucuuguuugacagcacucA..........    2     1    JCK-2
.......................ggaucuuguuugacagcacucu..........    5     0    JCK-1
.......................ggaucuuguuugacagcacucu..........    6     0    JCK-2
.......................ggaucuuguuugacagcacucu..........    4     0    JCK-3
.......................ggaucuuguuugacagcacucucu........    2     0    JCK-2
........................gaucuuguuugacagcacu............    3     0    JCK-1
........................gaucuuguuugacagcacu............    1     0    LT-1
........................gaucuuguuugacagcacu............    3     0    LT-3
........................gaucuuguuugacagcacu............    14    0    LT-2
........................gaucuuguuugacagcacu............    1     0    JCK-2
........................gaucuuguuugacagcacuc...........    3     0    LT-3
........................gaucuuguuugacagcacuc...........    2     0    LT-1
........................gaucuuguuugacagcacucu..........    1     0    LT-1
........................gaucuuguuugacagcacucA..........    1     1    LT-1
........................gaucuuguuugacagcacucuc.........    1     0    JCK-3
........................gaucuuguuugacagcacucuc.........    1     0    JCK-1
```

```
                    Mature                                              Star
uccaucuuguucguuuacccucuggaucuuguuugacagcacucucuccuaucuuguuugacugaugaugaugggagugcugucgacaagaucuagaagguacugucgaauaagauacagauacggaggaaggaucaacuuuaccuacucucacacaaucuucuucauauucucuucaauuuuuauuuuuuuauuguucguauuuuuuuucccagauuagauuucccuguuaaucaccaugguauuaaca
........................gaucuuguuugacagcacucuc......    4    0    JCK-2
.........................aucuuguuugacagcacu......    3    0    LT-2
.........................aucuuguuugacagcacu......    2    0    LT-1
.........................aucuuguuugacagcacuc......    8    0    LCK-3
.........................aucuuguuugacagcacuc......    1    0    LT-2
.........................aucuuguuugacagcacuU......    1    1    LT-2
.........................aucuuguuugacagcacuc......    1    0    JCK-2
.........................aucuuguuugacagcacuc......    5    0    LT-3
.........................aucuuguuugacagcacuc......    2    0    LT-1
.........................aucuuguuugacagcacuUu......    1    1    LT-3
.........................aucuuguuugacagcacucu......    24    0    LCK-3
.........................aucuuguuugacagcacucu......    4    0    LT-3
.........................aucuuguuugCcagcacucu......    1    1    JCK-2
.........................aucuuguuugacagcacucu......    15    0    JCK-2
.........................aucuuguuugacagcacucA......    1    1    LT-2
.........................aucuuguuugacagcacuUu......    1    1    LT-1
.........................aucuuguuugacagcacucu......    3    0    JCK-1
.........................aucuuguuugacagcacucA......    2    1    LT-1
.........................aucuuguuugacagcacucu......    4    0    JCK-3
.........................aucuuguuugacagcacucA......    7    1    LCK-3
.........................aucuuguuugacagcacucu......    2    0    LT-2
.........................aucuuguuugacagcacucu......    4    0    LT-1
.........................aucuuguuugacagcacucuc......    12    0    JCK-2
.........................Cucuuguuugacagcacucuc......    1    1    JCK-2
.........................aucuuguuugacagcacucuU......    6    1    LCK-3
.........................aucuuguuugacagcacucuc......    8    0    JCK-1
.........................aucuuguuugacagcacucuA......    1    1    JCK-3
.........................aucuuguuugacagcacucuA......    3    1    JCK-1
.........................aucuuguuugacagcacucuc......    11    0    JCK-3
.........................aucuuguuugacagcacucuU......    4    1    LT-2
.........................aucuuguuugacagcacucucG......    1    1    JCK-1
.........................aucuuguuugacGgcacucucu......    1    1    JCK-3
.........................aucuuguuugacagcacucCcu......    1    1    JCK-2
.........................aucuuguuugCcagcacucucu......    1    1    JCK-1
.........................aucuuguuugacagcacucucu......    107    0    JCK-2
.........................aucuuguuugacagcacucCcu......    1    1    JCK-3
.........................aucuuguuugacagcacucucu......    1    0    LT-1
.........................Cucuuguuugacagcacucucu......    1    1    JCK-1
.........................aucuuguuugacagcacucCcu......    1    1    LT-3
.........................aucuuguuugacagcacucCcu......    1    1    JCK-1
.........................aucuuguuugacagcacucucu......    92    0    JCK-3
.........................aucuuguuugacagcacucucu......    70    0    JCK-1
.........................aucuuguuugacagcacucucuA......    1    1    JCK-1
.........................aucuuguuugacagcacucucuc......    1    0    JCK-3
.........................aucuuguuugacagcacucucuc......    1    0    JCK-1
.........................aucuuguuugacagcacucucuc......    1    0    JCK-2
.........................aucuuguuugacagcacucucuA......    1    1    JCK-2
.........................aucuuguuugacagcacucucucc......    1    0    LT-1
..........................ucuuguuugacagcacuc......    2    0    LT-3
..........................ucuuguuugacagcacuc......    1    0    LT-2
..........................ucuuguuugacagcacucu......    2    0    JT-2
..........................ucuuguuugacagcacucu......    2    0    LT-1
..........................ucuuguuugacagcacucA......    1    1    LT-3
..........................ucuuguuugacagcacucu......    2    0    JCK-2
..........................ucuuguuugacagcacucuc......    2    0    JCK-2
..........................ucuuguuugacagcacucuc......    1    0    JCK-3
..........................ucuuguuugacagcacucucu......    2    0    LT-3
..........................ucuuguuugacagcacucucu......    1    0    LT-1
..........................ucuuguuugacagcacucucu......    13    0    JCK-2
..........................ucuuguuugacagcacucucu......    6    0    JCK-3
..........................ucuuguuugacagcacucucu......    3    0    JCK-1
..........................ucuuguuugacagcacucucuc......    2    0    JCK-3
..........................ucuuguuugacagcacucucuc......    2    0    JCK-1
..........................ucuuguuugacagcacucucuc......    1    0    LT-1
..........................ucuuguuugacagcacucucuc......    4    0    JCK-2
..........................ucuuguuugacagcacucucucA......    1    1    LT-1
...............................uuugacagcacucucuccu......    2    0    LT-3
.......................................cacucucuccuaucuuguuu......    1    0    LT-3
............................................ucuccuaucuuguuugacugauga......    2    0    JCK-3
............................................ucuccuaucuuguuugacugaugaugaugg......    1    0    LT-3
```

Mature **Star**

uccaucuuguucguuuacccucuggaucuuguuugacagcacucucuccuaucuuguuugacugaugaugaugggagugcugucgacaagaucuagaagguacugucgaauaagauacagauacggaggaaggaucaacuuuaccuacucucacacaaucuucuucauauucucuucaauuuuuauuuuuuuauuguucguauuuuuuuucccagauuagauuucccuguuaaucaccaugguauuaaca

ccuaucuuguuugacugaugaug	1	0	LT-1
cuaucuuguuugacugaugau	1	0	LT-1
ucuuguuugacugaugaugau	2	0	LT-2
uuguuugacugaugaugaugggag	6	0	LCK-3
ugacugaugaugaugggagugcug	1	0	LT-3
cugaugaugaugggagugcugucgacaaga	5	0	LCK-1
cugaugaugaugggagugcugucgacaaga	2	0	LCK-2
gaugaugaugggagugcugu	1	0	JCK-1
gaugaugaugggagugcugu	1	0	JCK-2
gaugaugaugggagugcugucgacaagauc	1	0	LT-2
ugaugaugggagugcugucgac	1	0	LT-1
gaugaugggagugcugucgacaa	7	0	LCK-3
gaugaugggagugcugucgacaagauc	1	0	LT-1
augaugggagugcugucgaca	2	0	JCK-2
ugaugAgagugcugucgacaagauc	1	1	JCK-1
ugaugAgagugcugucgacaagauc	2	1	JCK-2
ugaugAgagugcugucgacaagauc	1	1	JCK-3
gaugggagugcugucgacaaga	1	0	LT-3
augggagugcugucgacaagauc	8	0	LCK-3
augggagugcugucgacaagauc	1	0	LT-2
augggagugcugucgacaagauc	1	0	JCK-1
augggagugcugucgacaagauc	1	0	JCK-2
ugggagugcugucgacaagauc	6	0	JCK-2
ugAgagugcugucgacaagauc	1	1	JCK-1
ugggagugcugucgacaagauc	1	0	LT-3
ugggagugcugucgacaagauc	1	0	JCK-3
ugggagugcugucgacaagauc	4	0	LT-1
ugAgagugcugucgacaagauc	1	1	JCK-2
ugggagugcugucgacaagaucu	1	0	JCK-3
gggagugcugucgacaagauc	19	0	LCK-2
gggagugcugucgacaagauc	6	0	LT-2
gAgagugcugucgacaagauc	2	1	JCK-1
gggagugcugucgacaagauc	26	0	LCK-1
gggagugcugucgacaagauc	3	0	LT-1
gggagugcugucgacaagauc	3	0	JCK-2
gggagugcugucgacaagauc	1	0	LCK-3
gggagugcugucgacaagauc	1	0	JCK-3
gggagugcugucgacaagauc	8	0	LT-3
gggagugcugucgacaagauc	4	0	JCK-1
Agagugcugucgacaagau	1	1	JCK-3
Agagugcugucgacaagau	22	1	JT-1
Agagugcugucgacaagau	1	1	JCK-1
Agagugcugucgacaagau	2	1	JCK-2
Agagugcugucgacaagauc	51	1	JCK-1
Agagugcugucgacaagauc	70	1	JCK-2
Agagugcugucgacaagauc	10	1	JT-1
Agagugcugucgacaagauc	55	1	JCK-3
ggagugcugucgacaagaucu	1	0	JCK-1
ggagugcugucgacaagaucu	2	0	JCK-3
ggagugcugucgacaagaucuag	2	0	JCK-3
ggagugcugucgacaagaucuagU	1	1	LT-3
ggagugcugucgacaagaucuagU	1	1	JCK-1
ggagugcugucgacaagaucuagC	3	1	LT-1
ggagugcugucgacaagaucuaga	1	0	LT-3
gagugcugucgacaagauc	8	0	JCK-2
gagugcugucgacaagauc	2	0	LT-3
gagugcugucgacaagauc	3	0	LT-2
gagugcugucgacaagauc	3	0	JCK-3
gagugcugucgacaagauc	9	0	JCK-1
gagugcugucgacaagaucC	2	1	JCK-1
gagugcugucgacaagaucC	8	1	JT-3
gagugcugucgacaagaucu	2	0	LT-3
gagugcugucgacaagaucC	5	1	JT-2
gagugcugucgacaagaucC	10	1	JCK-3
gagugcugucgacaagaucC	9	1	JCK-2
gagugcugucgacaUgaucu	1	1	LT-1
gagugcugucgacaagaucCa	8	1	JCK-3
gagugcugucgacaagaucCa	15	1	JCK-2
gagugcugucgacaagaucuG	1	1	LT-1
gagugcugucgacaagaucCa	8	1	JCK-1

Mature Star

```
uccaucuuguucguuuacccucuggaucuuguuugacagcacucucuccuaucuuguuugacugaugaugaugggagugcugucgacaagaucuagaagguacugucgaauaagauacagauacggaggaaggaucaacuuuaccuacucucacacaaucuucuucauauucucuucaauuuuuauuuuuuuauuguucguauuuuuuuucccagauuagauuucccuguuaaucaccaugguauuaaca
..........................................................................gagugcugucgacaagaucuag.......................................................................................................................................................... 2 0 JCK-2
..........................................................................gagugcugucgacaagaucuag.......................................................................................................................................................... 2 0 LT-3
..........................................................................gagugcugucgacaagaucuaga......................................................................................................................................................... 6 0 JCK-1
..........................................................................gagugcugucgacaagaucCaga......................................................................................................................................................... 44 1 JCK-3
..........................................................................gagugcugucgacaagaucuaga......................................................................................................................................................... 9 0 LT-1
..........................................................................gagugcugucgacaagaucuaga......................................................................................................................................................... 1 0 LT-3
..........................................................................gagugcugucgacaagaucuaga......................................................................................................................................................... 15 0 JCK-2
..........................................................................gagugcugucgNcaagaucuaga......................................................................................................................................................... 1 1 JCK-1
..........................................................................gagugcugucgacaagaucCaga......................................................................................................................................................... 72 1 JCK-2
..........................................................................gagugcugucgacaagaucuagU......................................................................................................................................................... 1 1 JCK-2
..........................................................................gagugcugucgacaagaucuagU......................................................................................................................................................... 1 1 JCK-3
..........................................................................gagugcugucgacaagaucCaga......................................................................................................................................................... 35 1 JCK-1
..........................................................................gagugcugucgacaagaucuaga......................................................................................................................................................... 7 0 JCK-3
..........................................................................gagugcugucgacaagaucuagaagg...................................................................................................................................................... 5 0 LCK-3
..........................................................................gagugcugucgacaagaucuagaagg...................................................................................................................................................... 1 0 LT-3
...........................................................................agugcugucgacaagauc............................................................................................................................................................. 1 0 LT-1
...........................................................................agugcugucgacaagauc............................................................................................................................................................. 2 0 LT-3
...........................................................................agugcugucgacaagauc............................................................................................................................................................. 2 0 LCK-3
...........................................................................agugcugucgacaagauc............................................................................................................................................................. 3 0 JCK-2
...........................................................................agugcugucgacaagauA............................................................................................................................................................. 1 1 JCK-3
...........................................................................agugcugucgacaagauc............................................................................................................................................................. 1 0 JCK-3
...........................................................................agugcugucgacaagaucu............................................................................................................................................................ 2 0 JCK-3
...........................................................................agugcugucgacaagaucC............................................................................................................................................................ 3 1 JCK-3
...........................................................................agugcugucgacaaAaucu............................................................................................................................................................ 2 1 LCK-3
...........................................................................agugcugucgacaagaucC............................................................................................................................................................ 7 1 JCK-2
...........................................................................agugcugucgacaagaucu............................................................................................................................................................ 3 0 LCK-3
...........................................................................agugcugucgacaagaucCa........................................................................................................................................................... 4 1 JCK-3
...........................................................................agugcugucgacaagaucua........................................................................................................................................................... 1 0 JCK-3
...........................................................................agugcugucgacaagaucCa........................................................................................................................................................... 9 1 JCK-2
...........................................................................agugcugucgacaagaucua........................................................................................................................................................... 1 0 LT-1
...........................................................................agugcugucgacaagaucua........................................................................................................................................................... 2 0 LT-3
...........................................................................agugcugucgacaagaucCa........................................................................................................................................................... 2 1 JCK-1
...........................................................................agugcugucgacaagaucuag.......................................................................................................................................................... 3 0 JCK-2
...........................................................................agugcugucgacaagaucCag.......................................................................................................................................................... 2 1 JCK-2
...........................................................................agugcugucgacaagaucuag.......................................................................................................................................................... 10 0 LT-2
...........................................................................agugcugucgacaagaucCag.......................................................................................................................................................... 5 1 JCK-1
...........................................................................agugcugucgacaagaucCag.......................................................................................................................................................... 4 1 JCK-3
...........................................................................agugcugucgacaagaucuag.......................................................................................................................................................... 2 0 LT-3
...........................................................................agugcugucgacaagaucuag.......................................................................................................................................................... 2 0 JCK-3
...........................................................................agugcugucgacaagaucuagU......................................................................................................................................................... 10 1 LCK-3
...........................................................................agugcugucgacaagaucuaga......................................................................................................................................................... 1 0 LT-3
...........................................................................agugcugucgacaagaucuagU......................................................................................................................................................... 1 1 JCK-3
...........................................................................agugcugucgacaagaucCaga......................................................................................................................................................... 5 1 JCK-1
...........................................................................agugcugucgacaagaucuagU......................................................................................................................................................... 1 1 JCK-1
...........................................................................agugcugucgacaagaucCaga......................................................................................................................................................... 1 1 JCK-3
...........................................................................agugcugucgacaagaucCaga......................................................................................................................................................... 8 1 JCK-2
...........................................................................agugcugucgacaagaucCagaa........................................................................................................................................................ 1 1 JCK-2
...........................................................................agugcugucgacaagaucuagaag....................................................................................................................................................... 1 0 JCK-2
...........................................................................agugcugucgacaagaucuagaag....................................................................................................................................................... 3 0 JCK-3
............................................................................Cugcugucgacaagaucu............................................................................................................................................................ 1 1 LCK-3
............................................................................gugcugucgacaagaucC............................................................................................................................................................ 16 1 JCK-1
............................................................................gugcugucgacaagaucu............................................................................................................................................................ 29 0 LT-3
............................................................................gugcugucgacaagaucu............................................................................................................................................................ 14 0 LT-2
............................................................................gugcugucgacaagaucC............................................................................................................................................................ 3 1 JT-2
............................................................................gugcugucgacaagaucC............................................................................................................................................................ 5 1 JT-3
............................................................................gugcugucgacaagaucu............................................................................................................................................................ 2 0 JT-3
............................................................................gugcugucgacCagaucu............................................................................................................................................................ 1 1 LCK-2
............................................................................gugcugucgacaagaucu............................................................................................................................................................ 24 0 JCK-2
............................................................................gugcugucgacaagaucu............................................................................................................................................................ 27 0 JCK-1
............................................................................gugcugucgacaagaucu............................................................................................................................................................ 25 0 JCK-3
............................................................................gugcugucgacaagaucu............................................................................................................................................................ 1 0 JT-1
............................................................................gugcugucgacaagaucu............................................................................................................................................................ 36 0 LCK-3
............................................................................gugcugucgacaagaucu............................................................................................................................................................ 1 0 LCK-1
............................................................................gugcugucgacaagaucu............................................................................................................................................................ 21 0 LT-1
............................................................................gugcugucgacaagauUu............................................................................................................................................................ 2 1 JCK-1
............................................................................gugcugucgacaagaucC............................................................................................................................................................ 42 1 JCK-2
............................................................................gugcugucgacaagaucC............................................................................................................................................................ 18 1 JCK-3
............................................................................gugcugucgacaagaucu............................................................................................................................................................ 1 0 LCK-2
............................................................................gugcugucgacaagaucua........................................................................................................................................................... 25 0 LT-3
............................................................................Augcugucgacaagaucua........................................................................................................................................................... 1 1 LT-2
```

Mature (red) — **Star** (purple)

uccaucuuguucguuuacccucuggaucuuguuugacagcacucucuccuaucuuguuugacugaugaugauggagugcugucgacaagaucuagaagguacugucgaauaagauacagauacggaggaaggaucaacuuuaccuacucucacacaaucuucuucauauucucuucaauuuuuauuuuuuuauuguucguauuuuuuuucccagauuagauuucccuguuaaucaccaugguauuaaca

gugcugucgacaagaucua	4	0	JCK-2
gugcugucgacaagaucCa	9	1	JCK-3
gugcugucgacaagaucua	2	0	JCK-3
gugcugucgacaagaucua	10	0	LT-1
gugcugucgacaagaucua	14	0	LCK-2
gugcugucgCcaagaucua	1	1	LT-2
gugcugucgacaagaucCa	27	1	JCK-2
gugcugucgacaagaucCa	11	1	JCK-1
gugcugucgacaagaucCa	7	1	JT-1
gugcugucgacaagaucua	23	0	LT-2
gugcugucgacaagaucCa	8	1	JT-3
gugcugucgacaagaucua	7	0	JCK-1
gugcugucgacaagaucua	36	0	LCK-3
gugcugucgacaagaucua	23	0	LCK-1
gugcugucgacaagaucuU	10	1	LCK-3
gugcugucgacaaNaucua	1	1	LCK-1
gugcugucgacaagaucCa	7	1	JT-2
gugcugucgacaagaucuag	23	0	LT-2
gugcugucgacaagaucCag	9	1	JT-2
gugcugucgacaagaucuaA	1	1	LT-1
gugcugucgacaagaucCag	11	1	JCK-3
gugcugucgacaagaucuag	14	0	JCK-3
gugcugucgacaagaucuag	24	0	JCK-1
gugcugucgacaagaucCag	9	1	JT-3
gugcugucgacaagaucuag	39	0	LT-3
gugcugucgacaagaucuag	11	0	LT-1
guUcugucgacaagaucuag	1	1	LT-3
gugcugucgacaagaucuag	15	0	LCK-3
gugcugucgacaagaCcuag	1	1	LT-3
gugcugucgacaagaucuag	33	0	JCK-2
gugcugucgacaagaucCag	32	1	JCK-2
gugcugucgacaagaucCag	7	1	JCK-1
gugcugucgacaagaucGaga	1	1	JCK-1
guNcugucgacaagaucuaga	1	1	LCK-1
gugcugucgCcaagaucuaga	1	1	LCK-2
gugcugucgCcaagaucuaga	3	1	JCK-1
gugcugucgacaUgaucuaga	1	1	JCK-3
gugcugucgacaagaucCaga	1129	1	JCK-2
gugcugucgacaagaucuaga	5	0	JT-2
gugcugucgacNagaucuaga	1	1	JCK-1
gugcugucgacaagaucuaga	348	0	JCK-2
gugcugucgacaaAaucuaga	1	1	JCK-3
gugcugucgacaagaucuaga	158	0	LCK-2
gugcugucgacaagaucuagG	1	1	JCK-2
gugcugucgacaagCucuaga	1	1	JCK-3
gugcugucgacaCgaucuaga	1	1	LT-3
gugcugucgacaagGucuaga	1	1	LCK-3
gugcugucgacaagaucuaga	153	0	LT-2
gugcCgucgacaagaucuaga	1	1	LCK-1
gugcugucgacaagaucuaga	113	0	LCK-3
gugcugucgacaUgaucuaga	4	1	JCK-2
gugcugucgacaagCucuaga	1	1	JCK-1
gugcugucgCcaagaucuaga	1	1	JCK-2
gugUugucgacaagaucuaga	1	1	LT-3
gugcugucgacaagaucuaga	9	0	JT-3
gugcugucgacaGgaucuaga	1	1	LT-3
gugcugucgacaagaucCaga	22	1	JT-3
gugcugucgacaagaucuNga	1	1	JCK-1
gugcugucgacaagaucCaga	910	1	JCK-3
gugcugucgacaCgaucuaga	1	1	LCK-2
gugcugucgacaagaucuagU	7	1	JCK-2
gugcugucgacaagaucCaga	19	1	JT-1
Augcugucgacaagaucuaga	2	1	JCK-2
gugcugucgacaagaucuaga	238	0	LT-3
gugcugucgacaagaucuaga	191	0	JCK-1
gugUugucgacaagaucuaga	1	1	JCK-3
gugcugucgacaagaucuaga	169	0	LT-1
gugcugucgacaagaucCaga	18	1	JT-2
gugcugucgacaagaucuagU	5	1	LT-1
gugcugucgacaaAaucuaga	2	1	JCK-2

Mature **Star**

uccaucuuguucguuuacccucuggaucuuguuugacagcacucucuccuaucuuguuugacugaugaugaugggagugcugucgacaagaucuagaagguacugucgaauaagauacagauacggaggaaggaucaacuuuaccuacucucacacaaucuucuucauauucucuucaauuuuuauuuuuuuauuguucguauuuuuuuucccagauuagauuucccuguuaaucaccaugguauuaaca			
....gugcugucgacaUgaucuaga...	2	1	JCK-1
....gugcugucgacaagaucuagU...	3	1	JCK-1
....gugcugucgacaagaucuagU...	4	1	JCK-3
....gugUugucgacaagaucuaga...	2	1	JCK-1
....Uugcugucgacaagaucuaga...	1	1	JCK-2
....gugcugucgacaagaucuaga...	265	0	JCK-3
....gugcugucgacaagaucuagU...	8	1	LT-3
....gugcugucgacaagaucuaga...	167	0	LCK-1
....gugcugucgacaagaucCaga...	712	1	JCK-1
....gugcugucgacaagaucCagaa..	7	1	JCK-2
....gugcugucgacaagaucuagaU..	1	1	JCK-1
....gugcugucgacaagaucCagaa..	2	1	JCK-3
....gugcugucgacaagaucuagaa..	2	0	LT-1
....gugcugucgacaagaucCagaa..	6	1	JCK-1
....gugcugucgacaagaucuagaU..	2	1	JCK-3
....gugcugucgacaagaucuagaa..	5	0	LT-2
....gugcugucgacaagaucuagaaA...	3	1	LCK-3
....gugcugucgacaagaucuagaag...	2	0	JCK-1
....gugcugucgacaagaucuagaag...	1	0	JCK-2
....gugcugucgacaagaucuagaag...	1	0	JCK-3
....gugcugucgacaagaucuagaaA...	2	1	LT-3
....gugcugucgacaagaucuagaagg..	4	0	LCK-3
....gugcugucgacaagaucuagaagg..	1	0	LT-1
....ugcugucgacaagaucua..	12	0	LT-2
....ugcugucgacaagaucua..	31	0	LCK-3
....ugcugucgacaagaucua..	1	0	JCK-1
....ugcugucgacaagaucua..	19	0	LT-3
....ugcugucgacaagaucua..	1	0	JCK-2
....ugcugucgacaagaucua..	4	0	LT-1
....ugcugucgacaagaucuag...	3	0	JT-3
....ugcugucgacaagaucuag...	12	0	LCK-3
....ugcugucgacaagaucuaA...	1	1	JCK-1
....ugcugucgacaagaucuag...	9	0	LT-3
....ugcugucgacaagaucuaA...	1	1	LT-3
....ugcugucgacaagaucuag...	1	0	JT-2
....ugcugucgacaagaucCag...	2	1	JCK-2
....ugcugucgacaagaucuag...	1	0	JCK-2
....ugcugucgacaagaucuag...	14	0	LT-1
....ugcugucgacaagaucCag...	2	1	JCK-3
....ugcugucgacaagaucuag...	2	0	JCK-1
....ugcugucgacaagaucCag...	2	1	JCK-1
....ugcugucgacaagaucuag...	2	0	LT-2
....ugcugucgacaagaucuag...	1	0	JCK-3
....ugcugucgacaagaucuaga..	156	0	LT-3
....uAcugucgacaagaucuaga..	1	1	LT-3
....ugcugucgacaagaucuaga..	127	0	LT-1
....ugcugucgacaagCucuaga..	1	1	JT-3
....ugcugucgacaagaucuCga..	1	1	JT-2
....ugcugucgacaagaucuaga..	51	0	JCK-1
....ugcugucgacaagaucuaga..	13	0	JT-2
....ugcugucgacaagaucCaga..	125	1	JCK-2
....ugcugucgCcaagaucuaga..	1	1	LT-2
....ugcugucgacaagaucuaga..	107	0	JCK-2
....ugcugucgacaagaucuaga..	20	0	JT-3
....ugcugucgacaagaucuaga..	150	0	LT-2
....ugcugucgacaUgaucuaga..	1	1	JCK-3
....ugcugucgacaagaucuagU..	7	1	LT-3
....ugcugucgacaagaucuCga..	1	1	LT-3
....ugNugucgacaagaucuaga..	2	1	LT-2
....ugcugucgacaagaucuaga..	94	0	LCK-3
....ugcugucgacaagaucCaga..	79	1	JCK-1
....ugcugucgaAaagaucuaga..	1	1	LT-2
....ugcugucgacaagaucuaga..	13	0	JT-1
....ugcugucgacaagaucCaga..	105	1	JCK-3
....ugcugucgacaagaucuaga..	69	0	JCK-3
....ugcugucgacCagaucuaga..	1	1	LT-1
....ugcugucgacaagaucCaga..	20	1	JT-3
....ugcugucgCcaagaucuaga..	1	1	JCK-3
....uAcugucgacaagaucuaga..	2	1	LT-2
....ugcugucgacaagaucCaga..	13	1	JT-2

```
                    Mature                                              Star
uccaucuuguucguuuacccucuggaucuuguuugacagcacucucuccuaucuuguuugacugaugaugaugggagugcugucgacaagaucuagaagguacugucgaauaagauacagauacggaggaaggaucaacuuuaccuacucucacacaaucuucuucauauucucuucaauuuuuauuuuuuuauuguucguauuuuuuuucccagauuagauuucccuguuaaucaccaugguauuaaca
..............................................................................ugcugucgCcaagaucuaga	1	1	LT-3
..............................................................................ugcugucgacaagCucuaga	1	1	LT-1
..............................................................................ugcugucgacaagaucCaga	22	1	JT-1
..............................................................................ugcugucgacCagaucuaga	1	1	LT-3
..............................................................................ugcugucgacaagaucuCga	2	1	LT-2
..............................................................................ugcugucgacaagaucuagaU	3	1	LT-3
..............................................................................ugcugucgacaagaucuagaa	1	0	JCK-1
..............................................................................ugcugucgacaagaucCagaa	2	1	JCK-1
..............................................................................ugcugucgacaagaucuagaa	3	0	LT-1
..............................................................................ugcugucgacaagaucuagaU	3	1	JCK-3
..............................................................................ugcugucgacaagaucuagaU	1	1	JCK-2
..............................................................................ugcugucgacaagaucuagaa	3	0	JCK-2
..............................................................................ugcugucgacaagaucuagaU	6	1	LT-2
..............................................................................ugcugucgacaagaucuagaU	7	1	LT-1
..............................................................................ugcugucgacaagaucuagaa	1	0	LT-3
..............................................................................ugcugucgacaagaucuagaa	1	0	LT-2
..............................................................................ugcugucgacaagaucCagaa	1	1	JCK-3
..............................................................................ugcugucgacaagaucuagaagg	5	0	LT-3
..............................................................................ugcugucgacaagaucuagaagg	1	0	JCK-3
..............................................................................ugcugucgacaagaucuagaagg	2	0	JCK-2
..............................................................................ugcugucgacaagaucCagaaggu	1	1	JCK-2
...............................................................................gcugucgacaagaucuag	2	0	LT-2
...............................................................................gcugucgacaagaucuag	1	0	JCK-2
...............................................................................gcugucgacaagaucuaga	37	0	LCK-2
...............................................................................Ucugucgacaagaucuaga	1	1	JT-2
...............................................................................gcugucgacaagaucuaga	32	0	LCK-1
...............................................................................gcugucgacaagaucuaga	1	0	JT-2
...............................................................................gcugucgacaagaucuaga	4	0	LCK-3
...............................................................................gcugucgacaagaucuaga	4	0	LT-2
...............................................................................gcugucgacaagaucuaga	14	0	LT-3
...............................................................................gcugucgacaagaucuaga	3	0	JT-3
...............................................................................gcugucgacaagaucuaga	3	0	JCK-3
...............................................................................gcugucgacaagaucuaga	8	0	JCK-2
...............................................................................gcugucgacaagaucuaga	3	0	JCK-1
...............................................................................gcugucgacaagaucuaga	6	0	LT-1
...............................................................................gcugucgacaagaucuagaa	1	0	JCK-3
...............................................................................gcugucgacaagaucuagaa	1	0	JCK-2
...............................................................................gcugucgacaagaucuagaa	3	0	JT-1
...............................................................................gcugucgacaagaucuagaa	2	0	LT-3
...............................................................................gcugucgacaagaucuagaa	5	0	LT-1
...............................................................................gcugucgacaagaucCagaag	1	1	JCK-2
...............................................................................Ucugucgacaagaucuagaag	1	1	LT-3
...............................................................................gcugucgacaagaucuagaag	25	0	JCK-1
...............................................................................gcugucgacaagaucCagaag	4	1	JT-3
...............................................................................Acugucgacaagaucuagaag	1	1	LT-1
...............................................................................gcugucgacaagaucCagaag	3	1	JT-2
...............................................................................gcugucgacaagaucuagaag	2	0	JT-3
...............................................................................gcugucgacaagaucuagaag	12	0	LT-2
...............................................................................gcugucgacaagaucCagaag	3	1	JCK-3
...............................................................................gcugucgacaagaucCagaag	3	1	JCK-1
...............................................................................gcugucgacaagaucuagaag	18	0	JCK-3
...............................................................................gcugucgacaagaucuagaag	5	0	LCK-3
...............................................................................gcugucgacaagaucuagaag	11	0	JT-2
...............................................................................gcugucgacaagaucuagaag	17	0	LT-3
...............................................................................gcugucgacaagaucuagaag	9	0	LT-1
...............................................................................gcugucgacaagaucuagaag	21	0	JCK-2
...............................................................................gcugucgacaagaucuagaagAu	1	1	LT-1
...............................................................................gcugucgacaagaucuagaaggu	1	0	LT-2
................................................................................cugucgacaagaucuaga	1	0	LT-1
................................................................................cugucgacaagaucuaga	4	0	JT-2
................................................................................cugucgacaagaucuaga	6	0	LT-3
................................................................................cugucgacaagaucCaga	1	1	JCK-1
................................................................................cugucgacaagaucuaga	8	0	LCK-3
................................................................................cugucgacaagaucuaga	5	0	JT-3
................................................................................cugucgacaagaucuaga	1	0	JCK-1
................................................................................cugucgacaagaucCaga	1	1	JCK-2
................................................................................cugucgacaagaucuaga	12	0	LT-2
................................................................................cugucgacaagaucuagaa	3	0	LT-1
................................................................................cugucgacaagaucuagaa	3	0	LT-2
................................................................................cugucgacaagaucCagaa	1	1	JCK-3
```

```
                    Mature                                           Star
uccaucuuguucguuuacccucuggaucuuguuugacagcacucucuccuaucuuguuugacugaugaugaugggagugcugucgacaagaucuagaagguacugucgaauaagauacagauacggaggaaggaucaacuuuaccuacucucacacaaucuucuucauauucucuucaauuuuuauuuuuuuauuguucguauuuuuuuucccagauuagauuucccuguuaaucaccaugguauuaaca
...............................................................................cugucgacaagaucuagaa........................................................................................................................................................ 6 0 JT-3
...............................................................................cugucgacaagaucuagaa........................................................................................................................................................ 1 0 JCK-2
...............................................................................cugucgacaagaucuagaa........................................................................................................................................................ 2 0 JCK-3
...............................................................................cugucgacaagaucuagaa........................................................................................................................................................ 9 0 JT-2
...............................................................................cugucgacaagaucCagaa........................................................................................................................................................ 2 1 JCK-2
...............................................................................cugucgacaagaucCagaa........................................................................................................................................................ 2 1 JCK-1
...............................................................................cugucgCcaagaucuagaag....................................................................................................................................................... 1 1 LT-2
...............................................................................cugucgacaagaucuagaag....................................................................................................................................................... 18 0 LT-2
...............................................................................cugucgacaagaucCagaag....................................................................................................................................................... 1 1 JCK-1
...............................................................................cugucgacaagaucuagaaU....................................................................................................................................................... 1 1 JCK-3
...............................................................................cugucgacaagaucCagaag....................................................................................................................................................... 5 1 JCK-3
...............................................................................cugucgacaagaucuagaaC....................................................................................................................................................... 1 1 JCK-2
...............................................................................cugucgacaagaucuagaag....................................................................................................................................................... 3 0 JCK-1
...............................................................................cugucgCcaagaucuagaag....................................................................................................................................................... 1 1 LT-1
...............................................................................cugucgacaagaucuagaaU....................................................................................................................................................... 2 1 LT-1
...............................................................................cugucgacaagaucuagaag....................................................................................................................................................... 3 0 JCK-3
...............................................................................cugucgacaagaucuagaag....................................................................................................................................................... 7 0 LT-3
...............................................................................cugucgacaagaucuagaag....................................................................................................................................................... 6 0 LT-1
...............................................................................cugucgacaagaucCagaag....................................................................................................................................................... 4 1 JCK-2
...............................................................................cugucgacaagaucuagaagg...................................................................................................................................................... 16 0 LT-3
...............................................................................cugucgacaagaucCagaagg...................................................................................................................................................... 4 1 JCK-2
...............................................................................cugucgacaagaucuagaagA...................................................................................................................................................... 3 1 LCK-3
...............................................................................cugucgacaagaucuagaagg...................................................................................................................................................... 14 0 LT-2
...............................................................................cugucgacaagaucuagaagU...................................................................................................................................................... 2 1 LT-2
...............................................................................cugucgacaagaucuagaagg...................................................................................................................................................... 1 0 JCK-1
...............................................................................cugucgacaagaucuagaagg...................................................................................................................................................... 1 0 LT-1
...............................................................................cugucgacaagaucuagaagU...................................................................................................................................................... 1 1 LT-3
...............................................................................cugucgacaagaucuagaagU...................................................................................................................................................... 1 1 LT-1
...............................................................................cugucgacaagaucCagaagg...................................................................................................................................................... 3 1 JCK-3
...............................................................................cugucgacaagaucCagaagg...................................................................................................................................................... 5 1 JCK-1
...............................................................................cugucgacaagaucuagaagg...................................................................................................................................................... 12 0 LCK-1
...............................................................................cugucgacaagaucuagaagg...................................................................................................................................................... 4 0 JCK-2
...............................................................................cugucgacaagaucuagaagg...................................................................................................................................................... 7 0 JCK-3
...............................................................................cugucgacaagaucuagaagg...................................................................................................................................................... 22 0 LCK-2
...............................................................................cugucgacaagaucuagaaggu..................................................................................................................................................... 2 0 LT-1
...............................................................................cugucgacaagaucuagaagAu..................................................................................................................................................... 2 1 LT-2
...............................................................................cugucgacaagaucuagaagUu..................................................................................................................................................... 4 1 LCK-3
...............................................................................cugucgacaagaucuagaaggua.................................................................................................................................................... 1 0 LT-3
...............................................................................cugucgacaagaucuagaagguU.................................................................................................................................................... 10 1 LCK-3
................................................................................ugucgacaagaucCagaagg...................................................................................................................................................... 1 1 JCK-1
................................................................................ugucgacaagaucCagaaggu..................................................................................................................................................... 6 1 JCK-1
................................................................................ugucgacaagaucCagaaggu..................................................................................................................................................... 17 1 JCK-3
................................................................................ugucgacaagaucCagaaggu..................................................................................................................................................... 11 1 JCK-2
.................................................................................ucgacaagaucuagaaggu...................................................................................................................................................... 2 0 LT-2
.....................................................................................acaagaucuagaagguacugucgaaua.......................................................................................................................................... 1 0 LT-3
.....................................................................................acaagaucuagaagguacugucgaaua.......................................................................................................................................... 1 0 LT-1
...........................................................................................ucuagaagguacugucgaauaag........................................................................................................................................ 1 0 LT-3
...........................................................................................ucuagaagguacugucgaauaagaua..................................................................................................................................... 1 0 LT-2
...........................................................................................ucuagaagguacugucgaauaagauac.................................................................................................................................... 2 0 JCK-1
...........................................................................................ucuagaagguacugucgaauaagauac.................................................................................................................................... 1 0 JCK-3
...........................................................................................ucuagaagguacugucgaauaagauac.................................................................................................................................... 1 0 JCK-2
............................................................................................cuagaagguacugucgaauaagau...................................................................................................................................... 2 0 LT-2
.............................................................................................uagaagguacugucgaaua.......................................................................................................................................... 1 0 LT-2
..............................................................................................agaagguacugucgaauaagaua..................................................................................................................................... 1 0 LT-2
..............................................................................................agaagguacugucgaauaagaua..................................................................................................................................... 6 0 LCK-3
...............................................................................................gaagguacugucgaauaaga....................................................................................................................................... 2 0 LT-2
................................................................................................aagguacugucgaauaagauac.................................................................................................................................... 1 0 LT-2
.................................................................................................agguacugucgaauaagaua..................................................................................................................................... 3 0 LCK-3
.................................................................................................agguacugucgaauaagauac.................................................................................................................................... 4 0 LCK-3
......................................................................................................................agauacggaggaaggaucaacuuua........................................................................................................... 1 0 LT-1
........................................................................................................................auacggaggaaggaucaac............................................................................................................... 1 0 LT-1
.........................................................................................................................uacggaggaaggaucaacu.............................................................................................................. 2 0 LT-3
..............................................................................................................................................................................................uuauuguucguauuuuuuuuc....................................... 3 0 LT-2
.............................................................................................................................................................................................................uuuuucccagauuagauuuccc....................... 1 0 LT-3
```
